# Warming Reduces Parasitoid Success and Narrows Their Diet Breadth

**DOI:** 10.1111/ele.70322

**Published:** 2026-01-27

**Authors:** Chia‐Hua Lue, Mélanie Thierry, Leonardo Ré Jorge, Nicholas A. Pardikes, Megan Higgie, Jan Hrček

**Affiliations:** ^1^ Biology Centre of the Czech Academy of Sciences Institute of Entomology České Budějovice Czech Republic; ^2^ Department of Biology Hood College Frederick Maryland USA; ^3^ Faculty of Science University of South Bohemia České Budějovice Czech Republic; ^4^ Centre de Recherche sur la Biodiversité et l'Environnement (CRBE) UMR5300 CNRS‐IRD‐TINP‐UT3 Université Toulouse III—Paul Sabatier Toulouse France; ^5^ Department of Biology Utah State University Logan Utah USA; ^6^ College of Science and Engineering James Cook University Townsville Queensland Australia

**Keywords:** climate change, community, food web, redundancy, specificity, top‐down control

## Abstract

A significant area of current research is the impact of warming on trophic networks. However, few interactions per network are typically studied, which limits generalisation and precludes evaluation of impact on consumer diet breadth and redundancy of top‐down control. Here we show that experimental warming strongly decreased the success of parasitoid development across 28 *Drosophila‐*parasitoid interactions from a tropical rainforest network. Parasitoids responded consistently despite deep evolutionary divergence. Moreover, warming strongly narrowed the diversity of hosts that the parasitoids could use. Host developmental success was much less affected. In contrast, experimental cooling had only a mild effect on parasitoids and hosts. Our findings suggest that the top‐down control exerted by parasitoids is likely to weaken due to warming. The range of hosts that parasitoids can use will become more limited, potentially threatening the sustainability of parasitoid populations and changing the balance between trophic levels.

## Graphical abstract

Warming may have a major impact on trophic networks, but few interactions are typically studied. Here we show that experimental warming strongly decreased the success of parasitoid development across 28 *Drosophila*‐parasitoid interactions from a tropical rainforest network. Warming strongly narrowed the diversity of hosts that the parasitoids could use. The top‐down control exerted by parasitoids is thus likely to weaken due to warming and change the balance between trophic levels.

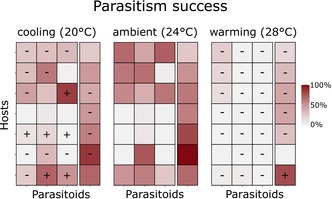

## 1 Introduction

Species interactions play key roles in trophic networks, providing functions such as top‐down control through predation and parasitism. The impact of environmental change on trophic networks and their functioning will thus largely depend on how the interactions are affected (Boukal et al. 2019; Gilman et al. 2010; Sentis et al. 2017; Tylianakis et al. 2008). Ecological networks typically encompass many species interactions (König et al. 2022; Rasmann et al. 2014), but experimental studies typically explore only a few interactions at a time due to feasibility (Malinski et al. 2024). However, identifying the impact of environmental changes on a diversity of interacting species is critical for understanding how general the response is within the community. Moreover, the impact on key aspects of trophic interactions, such as consumer diet breadth and redundancy of top‐down control, can only be evaluated from larger sets of interactions and is therefore poorly understood (Hallam and Harris 2023).

Ectotherms, such as insects, are strongly affected by global temperature changes (González‐Tokman et al. 2020; Harvey et al. 2020). Their thermal performance curves are known to be left‐skewed, so warming is expected to have a more pronounced effect than cooling (González‐Tokman et al. 2020). However, it remains unclear whether consumers are differently affected by temperature changes than resource species. There are indications that higher trophic levels may be especially vulnerable to environmental changes because they have to withstand the cumulative effects of environmental changes on themselves and on their resource species (Derocles et al. 2018; Tylianakis and Binzer 2014; Voigt et al. 2003). However, data are scarce, particularly for the interacting life stages of consumer and resource species (Derocles et al. 2018).

The dietary specialisation of interactions is crucial for the dynamics of trophic networks. Broad diet breadth allows consumers to switch among resource species depending on their availability, thereby supporting the persistence of consumer populations (Valdovinos et al. 2010). However, environmental change can modify diet breadth (Bestion et al. 2019; Hallam and Harris 2023). Some resource species can become unavailable (Hu et al. 2024), or the consumer may not be able to utilise a resource, for example, because of increased metabolic cost (Jeffs and Lewis 2013). Such narrowing of diet breadth can reduce population persistence. When observing interactions in nature (Rasmann et al. 2014), it is often difficult to distinguish whether changes in diet breadth are due to shifted preferences or the inability to use a resource in certain conditions. Experiments revealing the fundamental ability to utilise resource species are therefore necessary. In addition to the diet breadth of individual species, the distribution of interactions throughout the network and their redundancy significantly impacts resilience (Biggs et al. 2020). In trophic networks with more specialised interactions, energy flows across fewer pathways, which alters stability and robustness to environmental changes and extinctions (Rooney et al. 2006). Networks with more specialised interactions may thus face greater risks in a changing world.

Here, we investigated how the success of consumer and resource species development, consumer diet breadth, and redundancy of top‐down control are impacted by experimental warming and cooling. We focused on parasitoids, a group of natural enemies that exercise strong top‐down control of host populations (Derocles et al. 2018; de Sassi and Tylianakis 2012). The primary focus of environmental change studies is usually warming, but some regions may become cooler (Cohen et al. 2021). We measured the outcomes of 28 interactions between seven *Drosophila* species and four parasitoid species within a tropical rainforest trophic network at ambient (24°C), warming (28°C), and cooling (20°C) temperatures. We hypothesize that warming will be more stressful than cooling because of the nature of temperature performance curves in ectotherms. Warming may further disproportionately affect parasitoid success because of their higher trophic level and narrow down their diet breadth.

## 2 Materials and Methods

### 2.1 Study System

**FIGURE 1:**
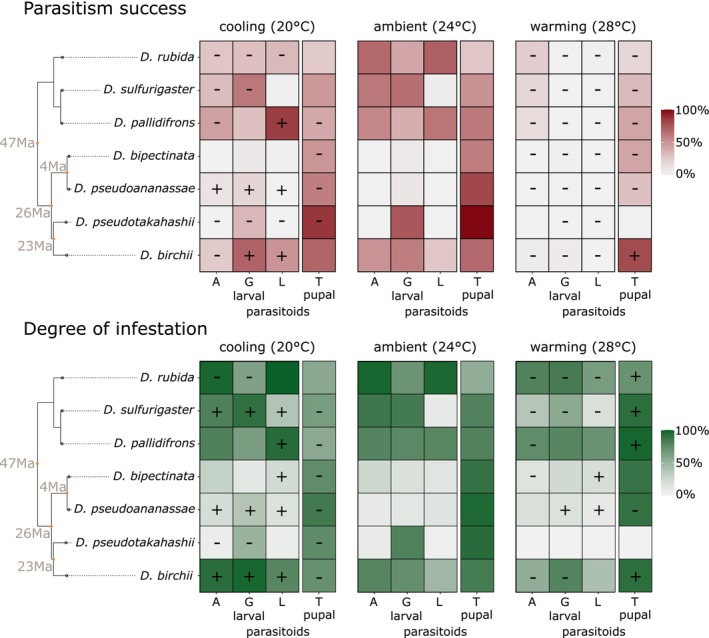
Mean parasitism success and degree of infestation for each host‐parasitoid interaction across temperature treatments. Host phylogeny is included, dating of nodes is from Suvorov et al. (2022). Larval parasitoids A: *Asobara* sp., L: *Leptopilina* sp., G: *Ganaspis* sp. Pupal parasitoid T: *Trichopria* sp. Significant parasitism success and degree of infestation changes with warming and cooling are marked with a sign: minus sign marks decrease from ambient temperature, plus sign marks an increase. Magnitude of the changes (contrasts) with confidence intervals is presented in Figure S1. Significance is considered as the contrasts whose 95% credible intervals are not overlapping with value 1. The ambient (24°C) treatment always serves as a base for the contrasts and significance compared to 20°C and 28°C; therefore, no significance is meaningful at 24°C. A separate model was run for larval and pupal parasitoids. Bayesian model summary is presented in Tables S1–S4.

The focal *Drosophila*‐parasitoid network represents a module of closely interacting species within the complex food web of the tropical rainforest of North Queensland, Australia (Jeffs et al. 2021). The network usually consists of ~5 host and ~5 parasitoid species per site (Jeffs et al. 2021). We focused on the seven most common *Drosophila* (listed in Figure 1) and four parasitoid species from the network. Three were larval parasitoids: *Asobara* sp. (Braconidae, strain KHB4, reference voucher USNMENT01557097), *Leptopilina* sp. (Figitidae, KH111F, USNMENT01557117), and *Ganaspis* sp. (Figitidae, KH69B, USNMENT01557100), and one was a pupal parasitoid *Trichopria* sp. (Diapriidae, 66LD, USNMENT01557254). For more details on the parasitoid strains see Lue et al. (2021). We established *Drosophila* and parasitoid cultures between 2017 and 2018 from Paluma (S18° 59.031′ E146° 14.096′) and Kirrama (S18° 12.134′ E145° 53.102′) sites (70–800 m a.s.l.), identified them using morphology and DNA barcoding (Jeffs et al. 2021), and shipped them to the Czech Republic under permit no. PWS2016‐AU‐002018 from the Australian Government, Department of the Environment. We maintained all cultures at 24°C on a 12:12 h light and dark cycle at Biology Centre CAS for ~25–50 generations before the experiments.

We kept *Drosophila* isofemale lines on standard *Drosophila* medium (corn flour, yeast, sugar, agar, and methyl‐4‐hydroxybenzoate). We combined five lines from each *Drosophila* species into a mass‐bred population to revive genetic variation before the experiment. Parasitoid lines were maintained on *Drosophila melanogaster* , which was not used in the experiment to avoid potential bias due to maternal effects.

### 2.2 Experimental Design

We aimed to reveal the impact of warming and cooling on the development of parasitoids, their hosts, and the feasibility of the interactions. In nature, host and parasitoid densities change widely due to the colonisation of new resource patches and linked population dynamics. To standardise our comparison, we fixed starting host density and parasitoid‐to‐host ratio in the experiment at low competition, based on preliminary assays and previous work (Thierry, Pardikes, Rosenbaum, et al. 2022), because high competition could reduce developmental success and make feasible interactions look unfeasible. We abbreviate the success of parasitoid development to ‘parasitism success’ in this paper. Parasitism success thus incorporates fitness components from fertile parasitoid females being presented with hosts (finding a host patch) and attacking them, to the emergence of adult parasitoids. Realised diet breadth in nature is likely to vary widely in time depending on the immediate availability of host species. We therefore measure the impact of warming and cooling on fundamental diet breadth from the tested set of hosts, that is, the diversity of hosts a parasitoid is able to utilise at a given temperature.

Three identical temperature control rooms simulated different thermal situations: cooling (20°C), ambient temperature (24°C), and warming (28°C) on a 12:12 h light cycle. The 24°C represents mean annual temperature in rainforest understory at low elevations in this host‐parasitoid community (Jeffs et al. 2021). The 20°C and 28°C are close to the lowest and highest daily average temperature of the natural environment (Jeffs et al. 2021). In a climate change model, a 1°C–5.7°C increase in temperatures by 2100 is predicted (IPCC 2023), thus 28°C represents a general warming scenario in tropical Queensland, Australia. According to temperature performance curves for part of the fly and parasitoid species studied here, the warming treatment is above their optimum and closer to their upper thermal limit, and thus reflects ecologically relevant stress (Bright et al. 2025; Chen and Lewis 2024).

We exposed each of the seven host species to all four parasitoid species (while maintaining unparasitized controls to assess host developmental success) at the three temperatures in a fully factorial design, replicated in 7–10 vials per treatment (depending on egg availability). We collected *Drosophila* eggs following an egg‐wash protocol adapted from (Nouhaud et al. 2018). The day before egg‐wash, we introduced two Petri dishes with agar gel topped with yeast paste in each population cage for one‐week‐old flies to lay eggs overnight. We then transferred 50 host eggs into 90 mm high and 28 mm diameter glass vials with 10 mL of media and directly placed the vials into the three different experimental temperatures. Altogether we collected a total of 49,950 eggs.

At 48 h for larval parasitoids and 120 h for the pupal parasitoid, we introduced three 3–6‐day‐old mated female parasitoids to each vial (except control vials). We removed the parasitoids 24 h later. Based on preliminary assays and published work, which included dissections of larvae for confirming parasitoid egg presence (Thierry, Pardikes, Rosenbaum, et al. 2022), these exposure conditions are sufficient for every host larva to be oviposited into at ambient, warming, and cooling temperatures, and the host stage is the most suitable for parasitoid development. We visually confirmed that oviposition occurred across all species and temperature combinations in the main experiment. We checked vials daily for adult emergence for up to 60 days. To avoid confounding counts with possible second generation, we stopped collection from a given vial after five consecutive days without emergence.

### 2.3 Parasitism Analysis

A parasitoid attack has three possible outcomes: wasp emerges, fly immune system fights off the infection and adult fly emerges, or both fly and wasp die. The interaction is characterised by a combination of parasitism success (success of parasitoid development, when wasps emerge) and degree of infestation (host suppression, when wasps emerge or both fly and wasp die). Both measures account for host control survival to separate the effect of parasitoids on host development from the effect of temperature. Parasitism success = *P*/*H*_*C*_ and degree of infestation = 1—(*H*/*H*_*C*_), where *P* is the number of parasitoid adults emerging from the parasitized vial, *H* is the number of adult hosts emerging from the parasitized vial, and *H*_*C*_ is the mean number of adult hosts emerging from the unparasitized vials at the given temperature (control). In some cases, *H*_*C*_ can be lower than *H* or *P*. We then take *H*_*C*_ to be the bigger of *H* or *P*, considering it the best estimate of the number of fly individuals that could have hatched in the vial if not exposed to parasitoids. The adjustment took effect in 15% of vials, negligibly modifying the distributions entering the Bayesian model. There was no systematic trend of this adjustment with temperature. Most cases occurred in *D. pseudoananassae* and *D. bipectinata* because of low parasitoid impact on those species and H thus more commonly exceeding Hc.

We built two sets of models: one including the three larval parasitoids and another including the pupal parasitoid to account for major life history differences. The immature stages of larval parasitoids develop within the host and come into direct contact with the host's immune system. In contrast, the adults of the pupal parasitoid *Trichopria* paralyse the host pupa, and the immature parasitoid develops on the outside of the pupa (but within the puparium).

We modelled the effects of temperature on parasitism success and degree of infestation using Bayesian multilevel regression models. The parasitism success and the degree of infestation were response variables, representing a binary outcome with a binomial distribution. For each host species and temperature, *H*_*C*_ was used as the number of trials in the Bayesian models. Then, for parasitism success, *P* was used as the outcome, while for degree of infestation we used *H*_*C*_—*H* as the outcome. We modelled temperature as a fixed effect. Additionally, host and parasitoid (only in larval parasitoid models) were included as random effects with an intercept and interaction with temperature represented as a categorical random slope, accounting for potential differences in temperature responses across hosts and parasitoids.

Phylogenetic divergence within *Drosophila* is deceptive. Although classified as a single genus, it encompasses widely divergent lineages that split some 47 million years ago (Suvorov et al. 2022). Such phylogenetic diversity in insects usually corresponds to subfamily or family taxonomic level (Blaimer et al. 2023). We therefore included hosts in the models both as a simple categorical random effect, which accounts for non‐phylogenetic variability, and to account for phylogeny also as a correlated random effect structured by the variance–covariance matrix from the *Drosophila* phylogeny. The phylogeny was taken and trimmed from a wider tree based on 1000 BUSCO genes (Kim et al. 2024), which is the most complete, but we added known approximate dating of nodes to figure 1 from (Suvorov et al. 2022). All models were fit using the R package *brms* (Bürkner 2017), using the default weakly informative priors for all parameters. For each model, eight chains were run in parallel, with 1000 iterations of warmup and 1250 iterations of usable samples, totaling 10,000 posterior samples.

To assess whether there was phylogenetic dependence, for each model we fitted an alternative model without the phylogenetic random effect and compared the two models using PSIS leave‐one‐out cross‐validation as implemented in the R package *loo* (Vehtari et al. 2024). In all cases, the models were not significantly different, but we conservatively present all results for the models with phylogeny to account for the intrinsic non‐independence of comparisons between species. Non‐phylogenetic results are shown in the supplement. Median posterior estimates for parasitism success and degree of infestation were extracted from the models for every temperature/host/parasitoid combination and contrasts of ambient (24°C) against cooling (20°C) and warming (28°C) were calculated as median odds ratios ±95% highest posterior density intervals using R package *emmeans* (Lenth 2024).

### 2.4 Host Developmental Success

We characterised the success of *Drosophila* host development at different temperatures using the proportion of emerging adult hosts from control vials not exposed to parasitoids. We applied analogous Bayesian analysis with temperature and host as predictors, using the number of eggs (50) as the number of trials and the number of emerging adults as the outcome.

### 2.5 Parasitoid Diet Breadth

The experimental setup of one‐on‐one, no‐choice assays measures fundamental diet breadth from the tested set of hosts, describing the diversity of hosts each parasitoid can develop on at a specific temperature. Realised diet breadth could be narrower (see Discussion). We characterised the parasitoid diet breadth by calculating the diversity of hosts from which each parasitoid species was successfully reared in a given set of replicates (Jorge et al. 2014). We constructed seven such sets for each combination of temperature and parasitoid species as each set had to include all studied host species and the minimum of replicates available was seven. We thus used the first seven replicates from each treatment. As the tests were carried out simultaneously, there was no a priori structure for assigning replicates to sets. In our main analysis, we thus grouped all first replicates into one diversity set, all second replicates into another, and continued this pattern until creating seven sets, reflecting the chronological preparation of the replicates. To assess the sensitivity of this approach, we randomised 1000 times the order of the replicates, checking what would be the observed diet breadths if the orders were completely arbitrary. The results were qualitatively the same.

Host diversity was calculated using alpha phylogenetic diversity based on Hill numbers (Chao et al. 2014) using package *hillR* (Li 2018). We set *q* = 1, which is equivalent to the Shannon diversity index, and we used the same host phylogeny described above to account for host relatedness. An alternative diet breadth metric disregarding host relatedness was also used, by calculating the same index with a star‐shaped phylogeny, but results did not qualitatively differ. Observed diet breadth in different sets was modelled by simple linear models, using temperature, parasitoid species and their interaction as predictors. Estimated marginal means and contrasts between ambient versus cooling and warming were extracted from the models using the *emmeans* R package. We employ the DSI* index to express phylogenetic specialisation relative to available host diversity (Jorge et al. 2014, 2017).

### 2.6 Additional Mechanisms

We conducted a number of supporting analyses with additional data from the experiment on fly and parasitoid body mass, parasitoid competition, and encapsulation. We report methods in the legends of the respective supporting information S1.

## 3 Results

### 3.1 Effect of Temperature on Host‐Parasitoid Interactions

Warming significantly and strongly decreased the parasitism success of larval parasitoids (Figure 1; Odds ratio (OR)_warming/ambient_ = 0.037 (95% credible interval 0.020–0.056)). An odds ratio of 0.037 means the odds of parasitism success in elevated temperatures is 27× less compared to ambient temperature. In the Bayesian models we used, a 95% credible interval for the odds ratio (OR) that does not include 1 indicates statistical significance. The parasitism success of the pupal parasitoid also significantly decreased with warming, but not as strongly (OR_warming/ambient_ = 0.466 (0.192–0.802)). Cooling significantly but weakly decreased the parasitism success of both larval and pupal parasitoids (OR_cooling/ambient_ = 0.828 (0.736–0.934) and 0.530 (0.423–0.633), respectively). There was expected variation in parasitism success between different species pairs at ambient and cooling temperatures (Figure 1; Figure S1, Tables S1 and S2). However, warming mostly removed this variation in larval parasitoids by strongly and consistently reducing parasitism success.

We used degree of infestation index as a measure of host suppression (Figure 1). Warming significantly but weakly decreased the degree of infestation by larval parasitoids (OR_warming/ambient_ = 0.710 (0.503–0.936)), and weakly increased the degree of infestation by the pupal parasitoid (OR_warming/ambient_ = 1.770 (1.016–2.687)). Cooling had an opposite effect and significantly weakly increased the degree of infestation by larval parasitoids (OR_cooling/ambient_ = 1.380 (1.259–1.507)), but decreased it in the pupal parasitoid (OR_cooling/ambient_ = 0.415 (0.351–0.483)). For details of contrasts and the model, see Figure S1 and Tables S3 and S4.

### 3.2 Effect of Temperature on Host Developmental Success

**FIGURE 2:**
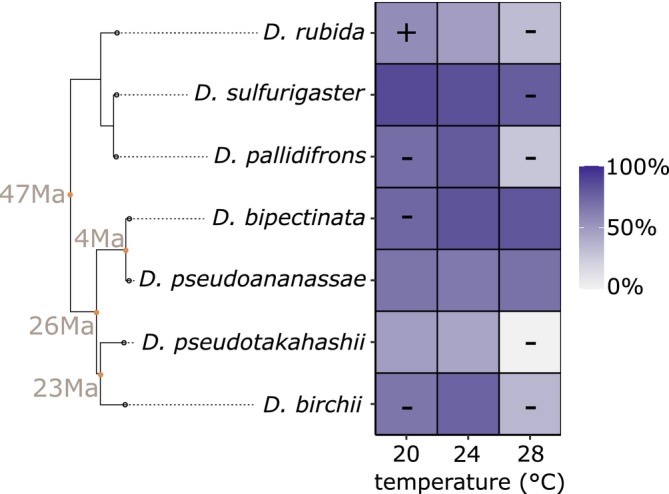
*Drosophila* host developmental success measured as mean survival from eggs to adults of the control group not subjected to infection in cooling (20°C), ambient (24°C), and warming (28°C) temperatures. Host phylogeny is shown, dating of nodes is from (Suvorov et al. 2022). Significant host developmental success changes with warming and cooling are marked with a sign: Minus sign marks decrease from ambient temperature, plus sign marks an increase. Magnitude of the changes (contrasts) with confidence intervals is presented in Figure S2. The ambient (24°C) treatment always serves as a base for the contrasts and significance compared to 20°C and 28°C; therefore, no significance is meaningful at 24°C. See also Table S5 for Bayesian model summary.

Success of host development in absence of parasitoids was significantly decreased by warming but not as strongly and uniformly as the parasitism success (Figure 2; Figure S2, Table S5, OR_warming/ambient_ = 0.198 (0.102–0.279)). The success of the development of five of the seven host species studied declined with warming, but the decline was a lot milder compared to parasitism success, which almost universally declined (compare odds ratios of warming on parasitoid and host developmental success; Figure S1 vs. Figure S2). Cooling did not have a significant effect on the success of host development in absence of parasitoids (OR_cooling/ambient_ = 0.928 (0.835–1.030)).

### 3.3 Effect of Temperature on Parasitoid Diet Breadth

**FIGURE 3:**
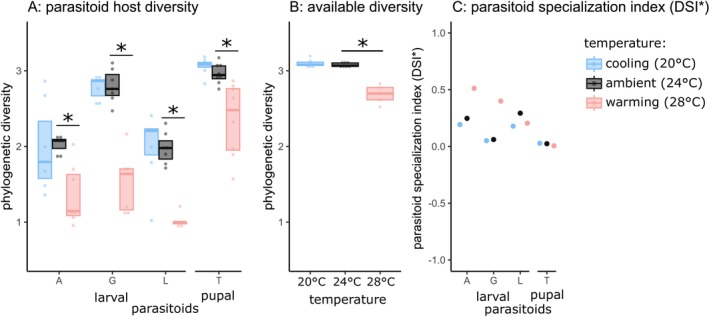
(A) Phylogenetic diversity of hosts successfully utilised by parasitoids decreases due to warming. Significant warming/ambient and cooling/ambient contrasts are marked with a star. (B) Available diversity of surviving hosts at each temperature. In (A) and (B), the phylogenetic diversity metric displayed is an equivalent of Shannon diversity taking phylogeny into account. The dots are observed values for each replicate, boxes represent the interquartile range, and the horizontal line represents the median of specialisation/availability values. (C) Parasitoid phylogenetic specialisation index relative to available host diversity abbreviated as DSI* (Jorge et al. 2014, 2017). The index ranges between −1 and 1, and a value of 0 means random diet, a positive value means higher specialisation relative to availability, and a negative value means more generality than would be expected based on availability (overdispersion). Separate models were run for larval and pupal parasitoids. For an analogous figure without phylogeny see Figure S7.

We quantified diet breadth as the diversity of hosts the parasitoids could utilise (successfully emerge from). Diversity of hosts that the parasitoids could utilise significantly decreased due to warming in all three larval parasitoids (Figure 3A; *Asobara* sp. estimate_warming/ambient_ = −0.66 ± 0.18, *p* < 0.001, *Ganaspis* sp. estimate_warming/ambient_ = −1.28 ± 0.18, *p* < 0.001, and *Leptopilina* sp. estimate_warming/ambient_ = −0.96 ± 0.18, *p* < 0.001), as well as the pupal parasitoid (*Trichopria* sp. estimate_warming/ambient_ = −0.61 ± 0.15, *p* = 0.001). Cooling did not significantly change the diversity of hosts utilised by larval parasitoids (*Asobara* sp. estimate_cooling/ambient_ = −0.05 ± 0.18, *p* = 0.925, *Ganaspis* sp. estimate_cooling/ambient_ = −0.02 ± 0.18, *p* = 0.989, and *Leptopilina* sp. estimate_cooling/ambient_ = 0.01 ± 0.18, *p* = 0.997), nor the pupal parasitoid (*Trichopria* sp. estimate_cooling/ambient_ = 0.10 ± 0.15, *p* = 0.738). Sensitivity analysis of distribution of replicates into diversity sets produced qualitatively the same results as the main analysis (Figure S3).

Diversity of parasitoid diet can be driven by diversity of hosts likely to be available (quantified as diversity of hosts that survive in a given temperature when not exposed to parasitism). Indeed, available diversity of surviving hosts significantly decreased due to warming (Figure 3B; estimate_warming/ambient_ = −0.39 ± 0.04, *p* < 0.001) but was not affected by cooling (estimate_cooling/ambient_ = 0.018 ± 0.04, *p* = 0.841). We therefore explored how successfully the parasitoids utilised host diversity in relation to availability. In the warming treatment, *Asobara* sp. and *Ganaspis* sp. were successfully utilising an even narrower range of hosts than what was available (Figure 3C). For *Leptopilina* sp. the difference was difficult to assess due to extremely low number of surviving individuals in warming (see Figure S4). If a parasitoid only rarely survives at a given temperature, measuring its diet breadth is difficult but also less important. The host diversity that the pupal parasitoid *Trichopria* sp. successfully utilised was very close to available diversity at all temperatures.

The results of non‐phylogenetic models were qualitatively the same as with phylogeny and we present them in the supplement (Figure S5–S7, Table S6–S10).

### 3.4 Host and Parasitoid Body Mass

Warming significantly reduced adult body mass the fly hosts acquired (Figures S8 and S9, Table S11). Parasitoid body mass correlated positively with host body mass at ambient temperature (Figure S10, Table S12), confirming that bigger hosts allow parasitoids to grow bigger. As few parasitoids hatched in warming, we could only measure body mass in nine cases. Warming significantly reduced body mass of *Asobara* sp. on all three hosts and of *Trichopria* sp. on three out of six hosts (Figure S11, Table S13).

## 4 Discussion

We examined how warming and cooling affect a wide range of host‐parasitoid interactions that are essential for the functioning of a trophic network. We found that parasitism success was strongly and consistently reduced by experimental warming. Importantly, warming restricted the diversity of hosts the parasitoid could utilise. Host developmental success was less affected by warming. In contrast, cooling had a weak impact on both parasitoids and hosts.

### 4.1 Low Parasitism Success due to Warming

The sweeping negative impact of warming on parasitism success in 25 of the 28 host‐parasitoid interactions studied provides strong evidence that global warming is likely to disrupt these interactions. The two cases with no detected change had no hosts or parasitoids hatched, which means the interaction was not possible under warming (Figure S4). Hosts were generally also affected by warming, but only three of the seven species strongly, two weakly and two not significantly impacted. The two trophic levels were therefore differentially affected. The negative impact of warming on parasitism is broadly consistent despite large differences in host immunity (Salazar‐Jaramillo et al. 2014) and parasitoid strategies to suppress immunity in the studied species (Prevost 2009). The evolutionary breadth of the studied interactions is considerable, as parasitoids represent deep evolutionary lineages that diverged more than 225 million years ago in the Triassic (Blaimer et al. 2023), and *Drosophila* host lineages split some 47 million years ago (Suvorov et al. 2022).

The impact of heat stress on parasitoids has to be understood in terms of dependence of their lifecycle on the hosts (Malinski et al. 2024). Particularly important is the larval stage exposed to combined effects of environmental changes on themselves and on their resource species. The negative impact of warming on parasitoid development we uncovered has been reported before but on only a few species pairs per study system (reviewed by Malinski et al. 2024; Thierry et al. 2019). However, several studies report the opposite trend of warming increasing the parasitism success (Thierry et al. 2019), and we also recorded a single such case in the pupal parasitoid *Trichopria* sp. attacking *D. birchii*. In the *Drosophila*‐parasitoid interactions we studied, parasitoids were more strongly impacted than the *Drosophila* because parasitism success was reduced more strongly than the degree of infestation. Other parts of the parasitoid lifecycle will likely amplify the negative effects of warming on parasitoid development. Pardikes et al. (2022) have shown that as host development speeds up with warming, the window of opportunity for parasitoids to attack the sensitive stage of the host will become narrower. Adult parasitoids may also be less heat tolerant than adult hosts (Furlong and Zalucki 2017; Wenda et al. 2023). Parasitoids will therefore likely be at an increasing disadvantage relative to their hosts with progressing climate change.

### 4.2 Narrower Parasitoid Diet Breadth

By measuring a large number of host‐parasitoid interactions, we uncovered a significant decrease in the diversity of hosts that the parasitoids were able to utilise with warming. In two species, the narrowing of diet goes beyond reduction in available host diversity. Three main factors can modify this reduction in diet breadth in nature: diet preferences and competition between parasitoids or hosts.

In a previous study, Thierry, Pardikes, Ximénez‐Embún, et al. (2022) found no significant preference of three parasitoid species on three *Drosophila* species. Behavioural preference and its changes due to warming can play a stronger role in more generalised predation systems, such as in spiders (Hu et al. 2024) or lizards (Bestion et al. 2019).

Competition between parasitoids is expected to decrease realised diet breadth further. With an additional experiment reported in the supplement, we found that parasitism success was lower in multi‐species than in single‐species infections in 17 out of 21 interactions tested, implying that competition can have strong negative effects (Figure S12 and Tables S14–S16). Interestingly, we also revealed an exceptional case of facilitation where *Leptopilina* sp. achieved higher parasitism success on *D. sulfurigaster* when co‐infecting with other parasitoids than when infecting alone (Figure S12). Attack of other parasitoids may have compromised host immunity, improving *Leptopilina* sp. development. The facilitation only occurred in cooling and ambient temperatures, not in warming. Competition between hosts can also modify parasitoid diet breadth. Specifically, Pardikes et al. (2022) showed that competition between hosts can render hosts suboptimal for parasitoid development.

### 4.3 Possible Mechanisms

The mechanisms behind the collapse of parasitism under warming are difficult to disentangle due to the intimacy of the interaction, but our results provide some insights. Both direct thermal stress on parasitoids and decline in host suitability may contribute to the observed patterns. Unsuitable hosts can be of low quality or have enhanced immunity. Reduced fly body mass with warming suggests flies became lower quality hosts, especially since we also detected a positive correlation between parasitoid and host body mass at ambient temperature. This is important because body mass is closely associated with reproductive success in parasitoids (Colinet et al. 2007). Few parasitoids hatched in warming, but parasitoid body mass seems more strongly affected by warming in the larval than the pupal parasitoid.

Larval parasitoids encounter host immunity while pupal parasitoids do not. The impact of warming on the parasitism success was stronger in larval parasitoids than in the pupal parasitoid *Trichopria* sp. *Trichopria* sp. even exerted significantly more pressure on the host in warming (measured by the degree of infestation). Narrowing of diet breadth was also more pronounced in larval parasitoids, while *Trichopria* sp. developed in hosts in proportion to their availability. Low performance in warming may thus be partially explained by living inside the host. However, more pupal parasitoids need to be studied to draw a general conclusion.

Immune response to parasitoids is extensively studied in *Drosophila melanogaster* and includes both cellular and humoral response (Lemaitre and Hoffmann 2007). In *Drosophila melanogaster* and close relatives, parasitoid egg is encapsulated by hemocytes, melanises, and the melanised capsules remain in emerging adult flies as evidence of successful defence (Salazar‐Jaramillo et al. 2014). We therefore screened flies of all species from the experiment for presence of melanised capsules. However, our data are inconclusive about the impact of warming on encapsulation. We only found encapsulation in *D. pseudotakahashii* (consistently with melanised encapsulation being phylogenetically restricted (Salazar‐Jaramillo et al. 2014)), and no fly adults of this species hatched in warming (Figure S13, Tables S17 and S18). Recent study of behavioural fever in *D. melanogaster* suggests that increased temperatures upregulate immunity, especially humoral production of antimicrobial peptides that damage the wasp microbiome (Sheng et al. 2025). However, it is unclear if this extends to the *Drosophila* species we studied.

Traits connected to oviposition such as cuticle hardness can also influence parasitism (Broski and King 2015). In our experiment a total of 13 out of 28 interactions show the negative impact of parasitoids on hosts in warming, either in increased degree of infestation (Figure 1) or in reduced adult fly body mass compared to unparasitized controls (Figure S14). Oviposition must have therefore taken place in warming and is unlikely to be driving the collapse of parasitism success in warming. Temperature can also influence the effectiveness of parasitoid venom, but evidence is very limited (Cavigliasso et al. 2021).

### 4.4 Consequences for Trophic Networks

The combination of a narrower diet breadth and lower parasitism success due to warming poses a serious threat to parasitoids. Parasitoids are among the most species‐rich groups of organisms, and lower persistence of their populations may lead to a loss of biodiversity. We performed a single‐generation experiment, and the dynamic consequences in nature can be complex. While high parasitism success does not necessarily guarantee better parasitoid population persistence, very low parasitism success unequivocally means low top down control and decreased parasitoid population persistence (Pardikes et al. 2022). Restricted diet breadth is likely to further decrease persistence. However, confirming the impact in natural communities is difficult as other mechanisms can influence the consequences. For example, *Asobara* sp. is capable of a certain level of acclimation (Bright et al. 2025). Although existing studies suggest that the possibility of thermal adaptation is likely to be limited, particularly in tropical species (Kellermann et al. 2012), local adaptation and demographic buffering cannot be excluded. These aspects are very little understood for parasitoids. Field observations in our study system show an increase in parasitism success from high elevations to low elevations (Jeffs et al. 2021). Based on mean annual temperatures, this qualitatively corresponds to the increase we found from cooling to ambient temperature. However, predicting the impact of warming beyond existing environmental gradients is very challenging. Interestingly, existing elevation gradient studies from the temperate region show a narrower diet breadth at the warm end of the gradient (Bonnaffé et al. 2024; König et al. 2022; Rasmann et al. 2014), but extrapolation to even warmer environments is unclear.

Due to lower parasitoid diet breadth and lower parasitism success, the host‐parasitoid networks impacted by warming are likely to consist of fewer but more specialised interactions and become less connected. This implies lower redundancy, when even a localised impact in the network is likely to lead to loss of function, for example regulation of a particular resource species (Biggs et al. 2020; Sanders et al. 2018). Other groups of natural enemies, like predators or pathogens, may compensate (Roslin et al. 2017), but evidence is lacking. If parasitoids are not compensated, resource species will be released from top‐down regulation with cascading effects until a new balance between the trophic levels in the network is reached. Herbivore populations, already benefiting from higher primary production (Tylianakis et al. 2008), are likely to increase further. The effectiveness of biological control in economically important systems could be reduced, as evidence from field trials shows (Derocles et al. 2018; Tylianakis and Binzer 2014).

## Author Contributions

Chia‐Hua Lue conceived the project; Mélanie Thierry, Nicholas A. Pardikes, and Jan Hrček contributed to the experimental design; Chia‐Hua Lue, Mélanie Thierry, and Nicholas A. Pardikes collected the data; Leonardo Ré Jorge, Mélanie Thierry, Nicholas A. Pardikes, and Jan Hrček analysed the data. Megan Higgie contributed to obtaining and establishing the experimental lines. Jan Hrček led the writing of the manuscript, and all authors contributed critically to the drafts and gave final approval for publication.

## Funding

This work was supported by Grantová Agentura České Republiky (20‐30690S).

## Conflicts of Interest

The authors declare no conflicts of interest.

## Supporting information


**Data S1:** Supporting Information.

## Data Availability

All data and R scripts used for this study were archived and are openly available in Zenodo https://doi.org/10.5281/zenodo.16317124 (Lue et al. 2025).
