# Supplementary material for: Warming Reduces Parasitoid Success and Narrows Their Diet Breadth
Source: Ecol Lett. 2026 Jan 27;29(1):e70322. doi: 10.1111/ele.70322 (PMC12836454; doi:10.1111/ele.70322)
Supplement: Supplementary file 1 — Data S1: Supporting Information. [file ELE-29-0-s001.pdf]

Supplementary information for:

Warming reduces parasitoid success and narrows their diet breadth

Chia-Hua Lue, Mélanie Thierry, Leonardo Ré Jorge, Nicholas A. Pardikes, Megan Higgie & Jan Hrček

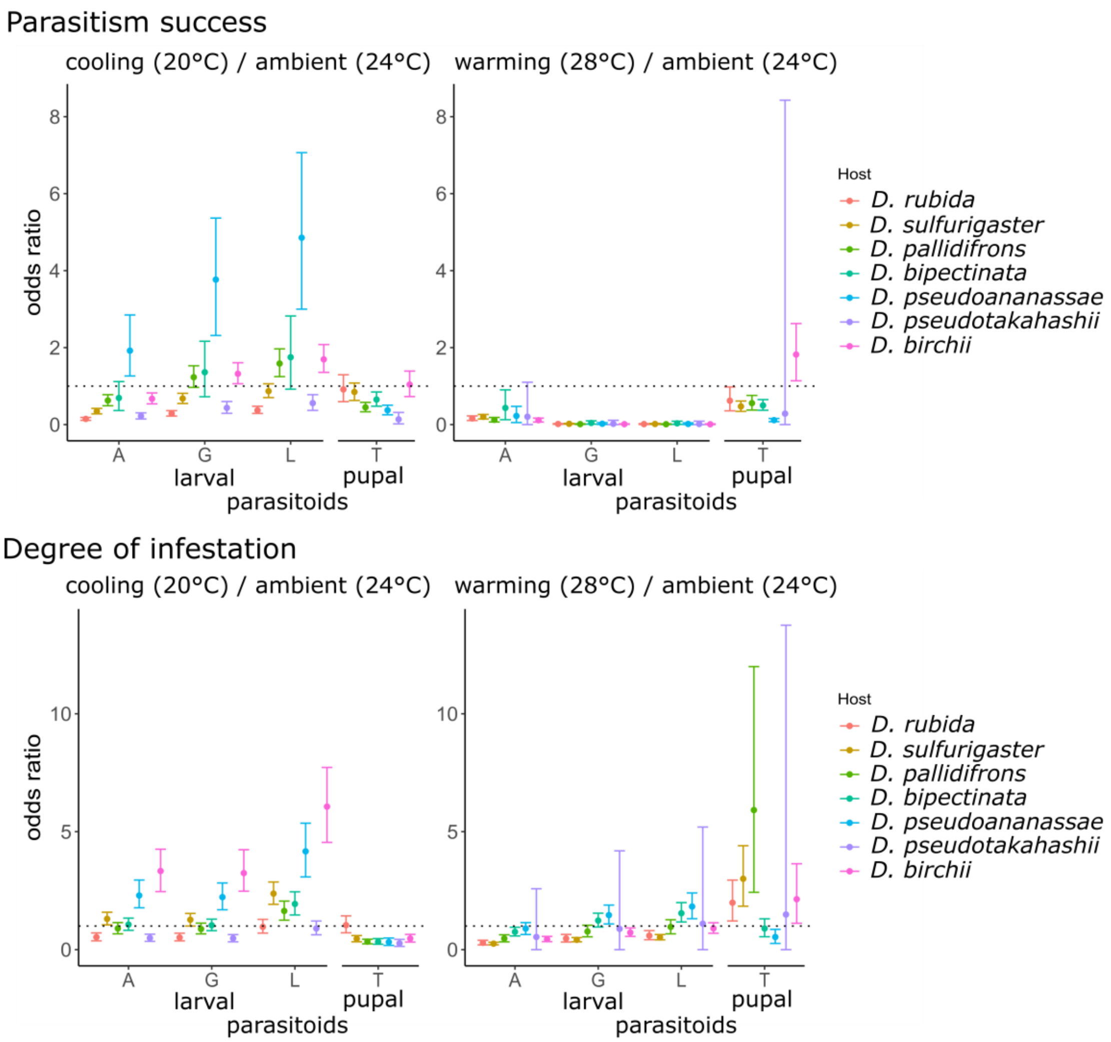

Figure S1. Thermal effect of cooling and warming on parasitism success (top) and degree of infestation (bottom) for each host – parasitoid combination. Values below the dotted line (lower than 1) mean better performance at ambient temperature, values above the line mean better performance in cooling or warming, respectively. When the 95% confidence interval shown does not include 1 the difference can be considered significant. Some confidence intervals are large as a result of small number of individuals hatching at the given temperature. Parasitoid species codes: A: *Asobara* sp., L: *Leptopilina* sp., G: *Ganaspis* sp., T: *Trichopria* sp. Contrasts for pupal parasitoid "T" come from a separate model, which is signified by interrupted x axis.

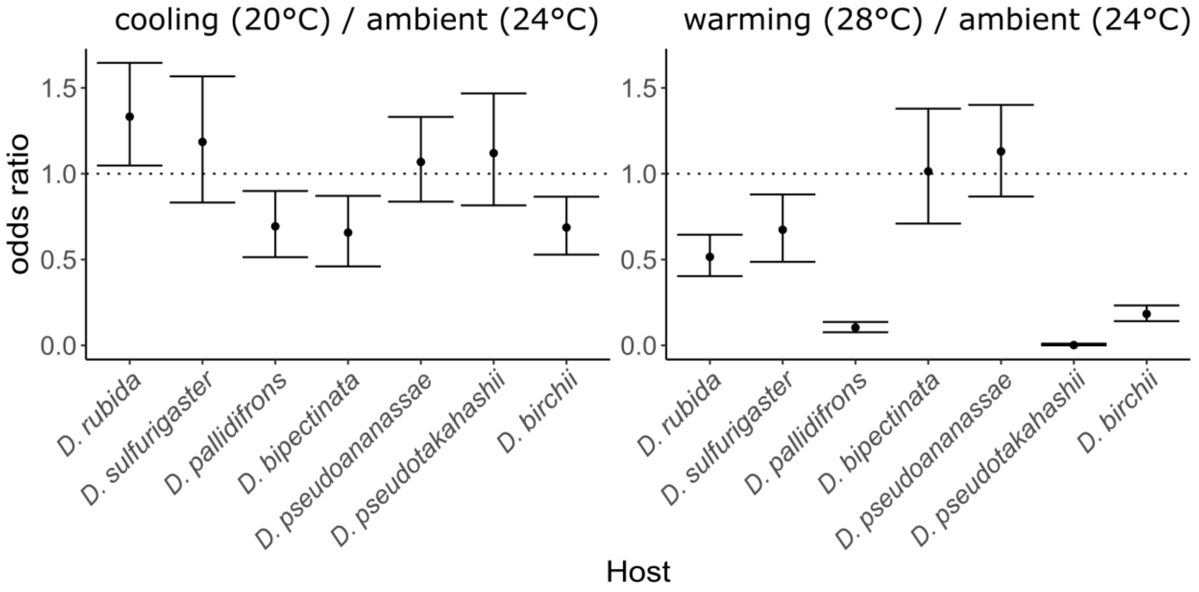

Figure S2. Thermal effect of cooling and warming on *Drosophila* host developmental success without infection. Values below the dotted line (lower than 1) mean better performance at ambient temperature, values above the line mean better performance in cooling or warming, respectively. When the 95% confidence interval shown does not include 1 the difference can be considered significant.

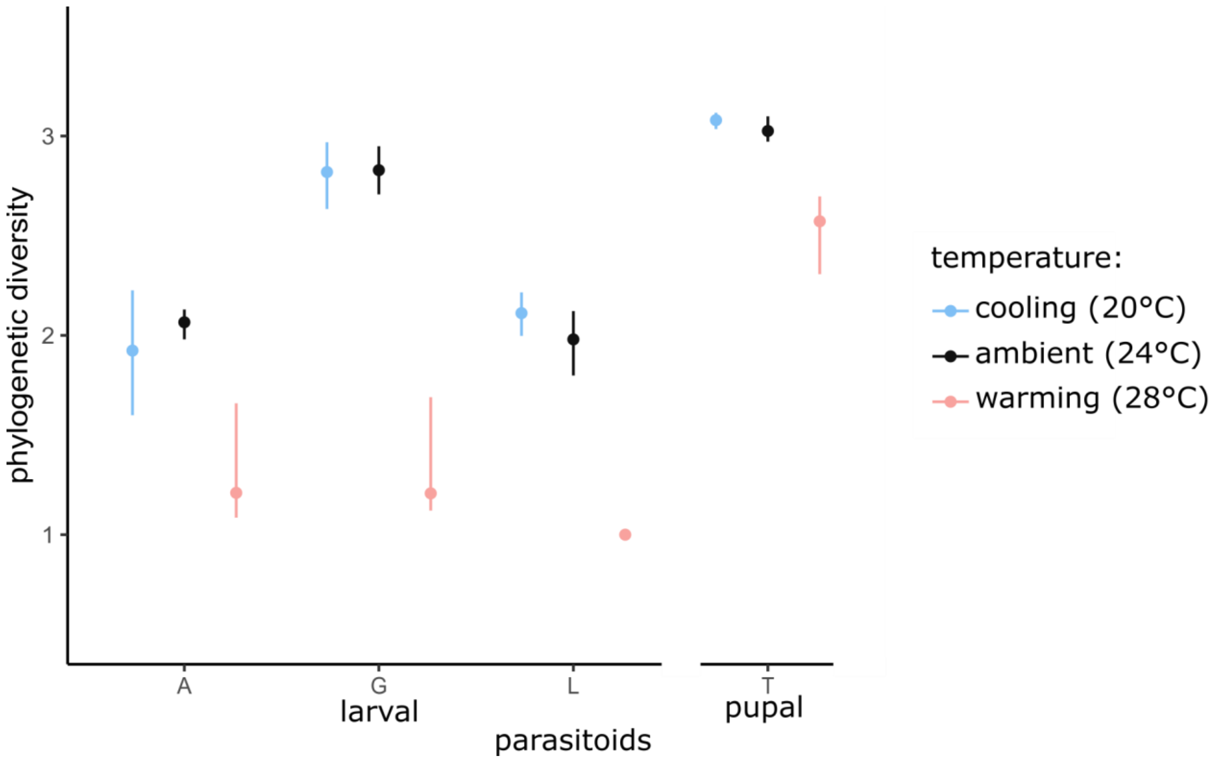

Figure S3. Sensitivity analysis of distribution of replicates into diversity sets. Diet breadth was measured as the diversity of hosts each parasitoid can develop on in a given temperature in one-on-one, no-choice assays (fundamental diet breadth). Figure 3A represents chronological arrangement of replicates into diversity sets. To assess the sensitivity of this approach, we present in this figure the result of median and interquartile range from 1000 randomizations of the order of the replicates, checking what would be the mean observed diet breadth across the seven diversity sets if the orders were completely arbitrary. Separate models were run for larval and pupal parasitoids. Note that the patterns and differences between parasitoids and temperatures match the observations in Figure 3A.

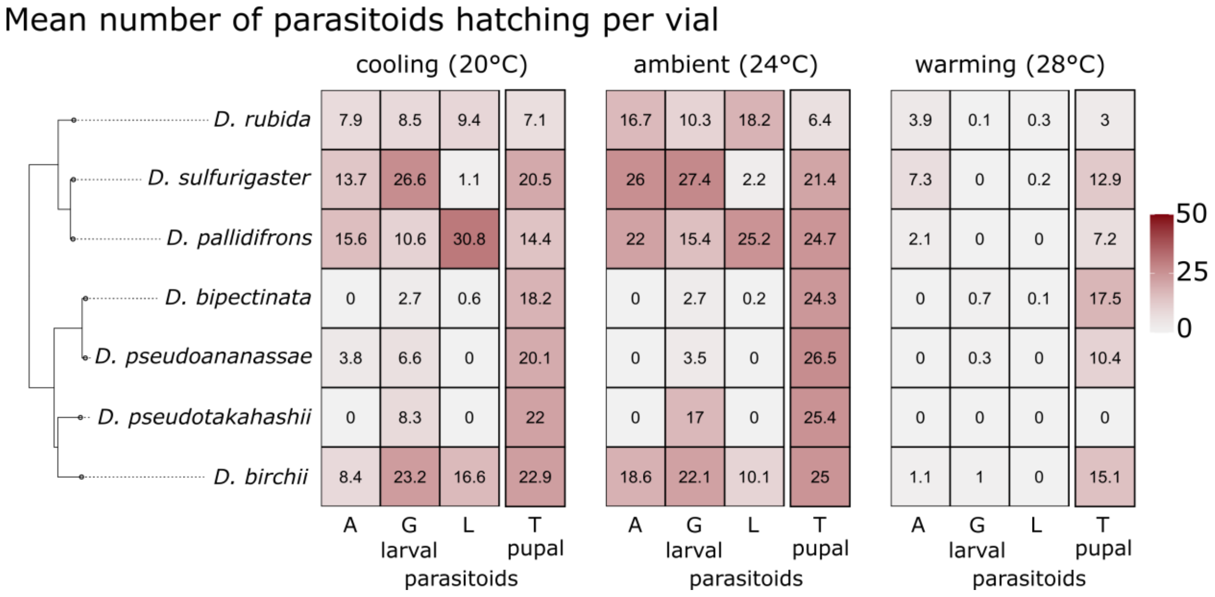

Figure S4. Mean number of parasitoids emerging per vial across host-parasitoid combinations and temperature treatments. This is modification of Fig. 1 with mean number of parasitoids instead of parasitism success. Mean is from seven replicate vials. Host phylogeny is included. Larval parasitoids A: *Asobara* sp., L: *Leptopilina* sp., G: *Ganaspis* sp. Pupal parasitoid T: *Trichopria* sp.

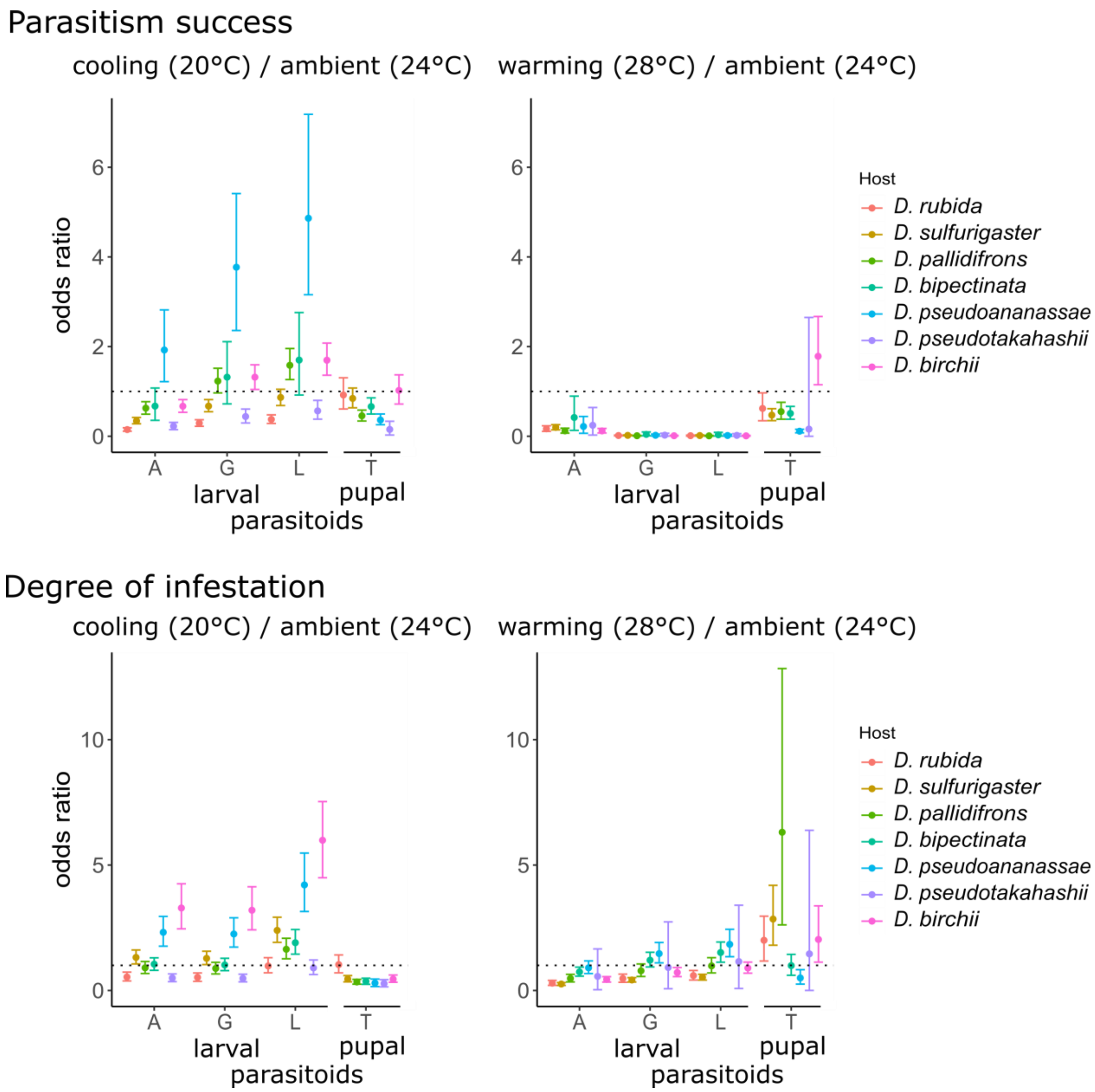

Figure S5. *This figure contains the same information as Fig. S1, but with a model fit without taking host phylogeny into account.* Thermal effect of cooling and warming on parasitism success (top) and degree of infestation (bottom) for each host – parasitoid combination. Values below the dotted line (lower than 1) mean better performance at ambient temperature, values above the line mean better performance in cooling or warming, respectively. When the 95% confidence interval shown does not include 1 the difference can be considered significant. Some confidence intervals are large as a result of small number of individuals hatching at the given temperature. Parasitoid species codes: A: *Asobara* sp., L: *Leptopilina* sp., G: *Ganaspis* sp., T: *Trichopria* sp. Contrasts for pupal parasitoid “T” come from a separate model, which is signified by interrupted x axis. See Tables S6-S9 for Bayesian model summaries.

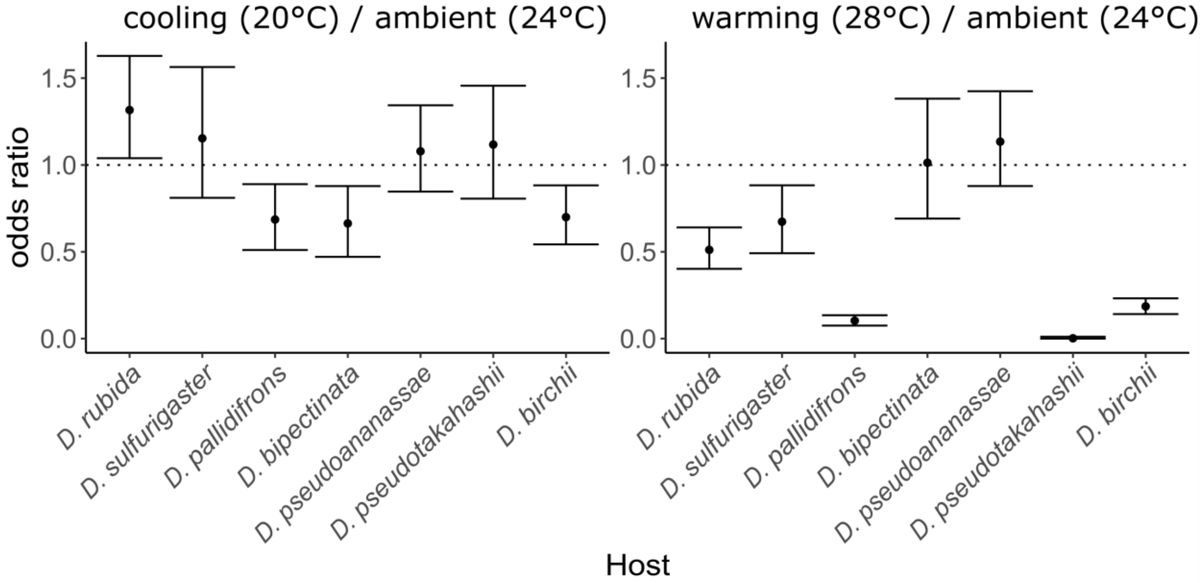

Figure S6. *This figure contains the same information as Fig. S2, but with a model fit without taking host phylogeny into account.* Thermal effect of cooling and warming on *Drosophila* host developmental success without infection. Values below the dotted line (lower than 1) mean better performance at ambient temperature, values above the line mean better performance in cooling or warming, respectively. When the 95% confidence interval shown does not include 1 the difference can be considered significant. See Table S10 for Bayesian model summary.

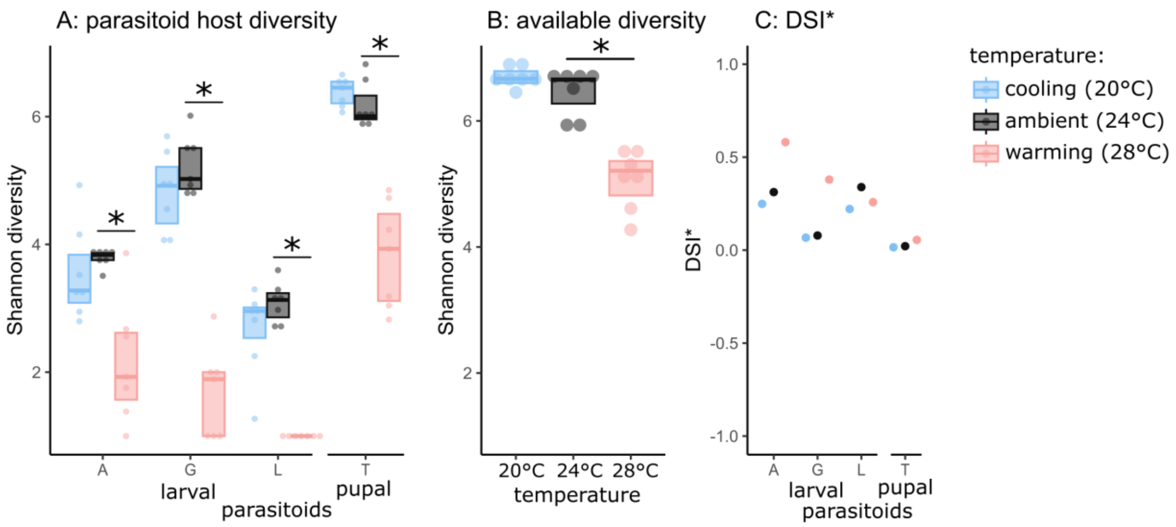

Figure S7. *This figure contains the same information as Fig. 3, but with Shannon diversity instead of phylogenetic diversity.* (A) Shannon diversity of hosts successfully utilized by parasitoids decreases due to warming. Significant warming/ambient and cooling/ambient contrasts are marked with a star. (B) Available Shannon diversity of surviving hosts at each temperature. In (A) and (B), the dots are observed values for each replicate, boxes represent the interquartile range, and the horizontal line represents the median of specialization/availability values. (C) Shannon specialization index relative to available host diversity (abbreviated as DSI*). The index ranges between -1 and 1, and value of 0 means random diet, positive value means higher specialization relative to availability, and negative value means more generality than would be expected based on availability (overdispersion). This is originally a phylogenetic index, but here it was computed on star phylogeny and therefore translates to Shannon diversity. Separate models were run for larval and pupal parasitoids.

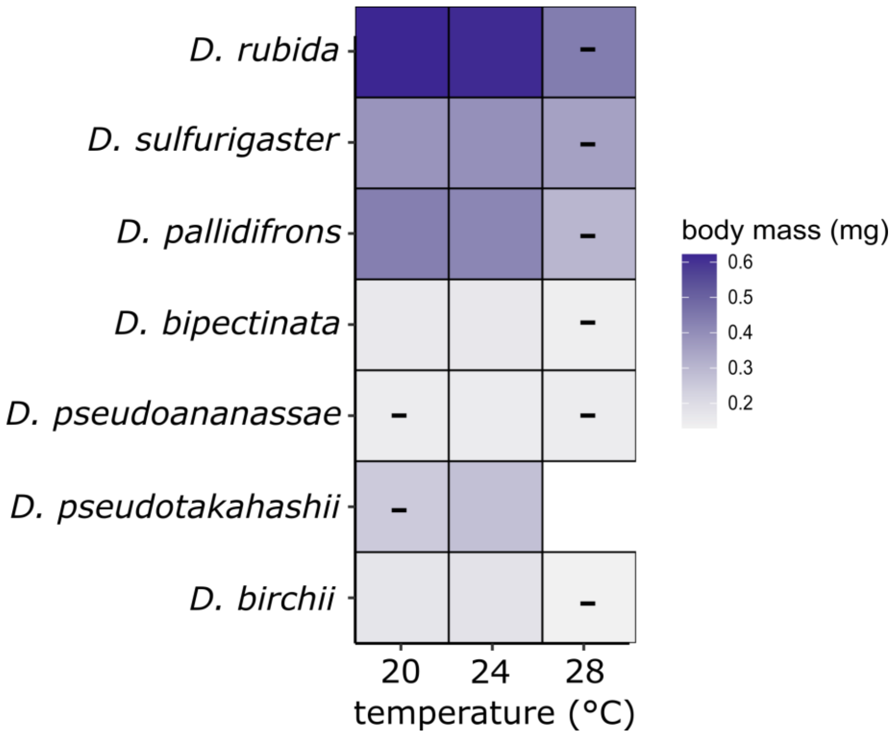

Figure S8. *Drosophila* host adult dry body mass of the flies hatching from the control group not subjected to infection in cooling (20°C), ambient (24°C), and warming (28°C) temperatures. Significant mass changes with warming and cooling are marked with a sign: minus sign marks decrease from ambient temperature, plus sign marks an increase. Magnitude of the changes (contrasts) with confidence intervals is presented in Fig. S9. The ambient (24°C) treatment always serves as a base for the contrasts and significance compared to 20°C and 28°C, therefore no significance is meaningful at 24°C. Here we present the estimates for control flies, obtained from a model for body mass of all flies measured. In this model, body mass was the response variable with a normal distribution, and predictors were temperature as a categorical fixed parameter and two grouping variables: parasitoid species (control and 4 parasitoids), with temperature as a group-level variable, and host with both parasitoid and temperature and group-level variables. Phylogeny was not used in this analysis and is thus not displayed. See Table S11 for Bayesian model summary. To measure dry body mass, we randomly selected 20 individuals for each combination of species and temperature, dried them in an oven and individually weighted them. No *D. pseudotakahashii* hatched in warming and there are thus no data for this combination.

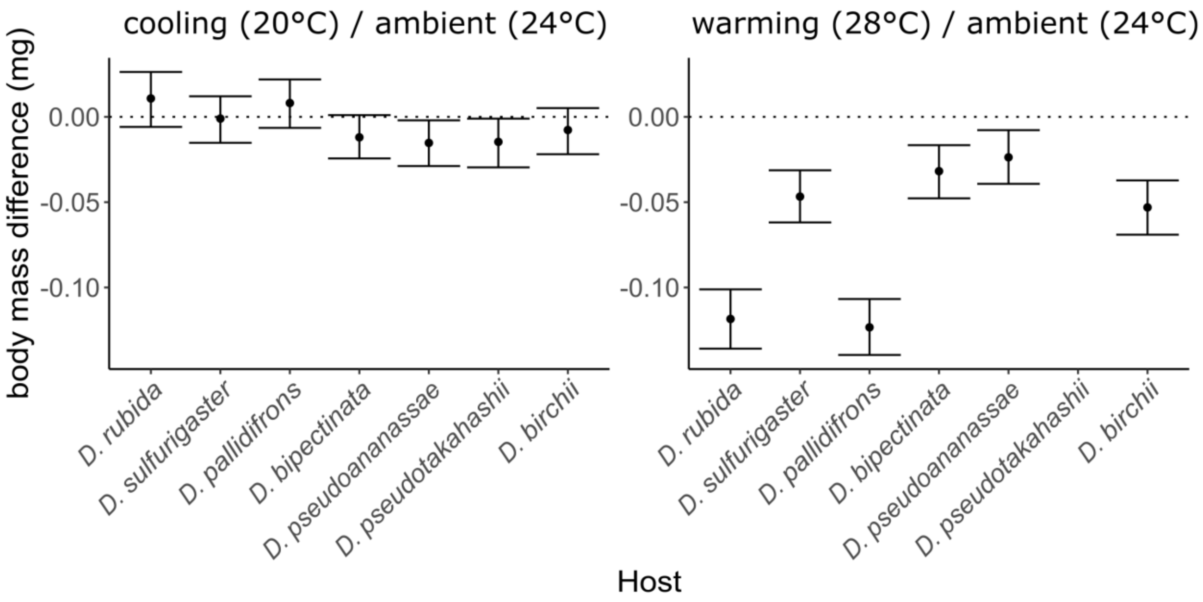

Figure S9. Thermal effect of cooling and warming on *Drosophila* host adult dry body mass of the flies hatching from the control group not subjected to infection. Values below the dotted line (lower than 0) mean higher body mass at ambient temperature, values above the line mean higher body mass in cooling or warming, respectively. When the 95% confidence interval shown does not include 0 the difference can be considered significant. There are no data for *D. pseudotakahashii* in warming as no adult flies hatched. Please refer to Figures S8 and Table S11 for further details on the models employed here.

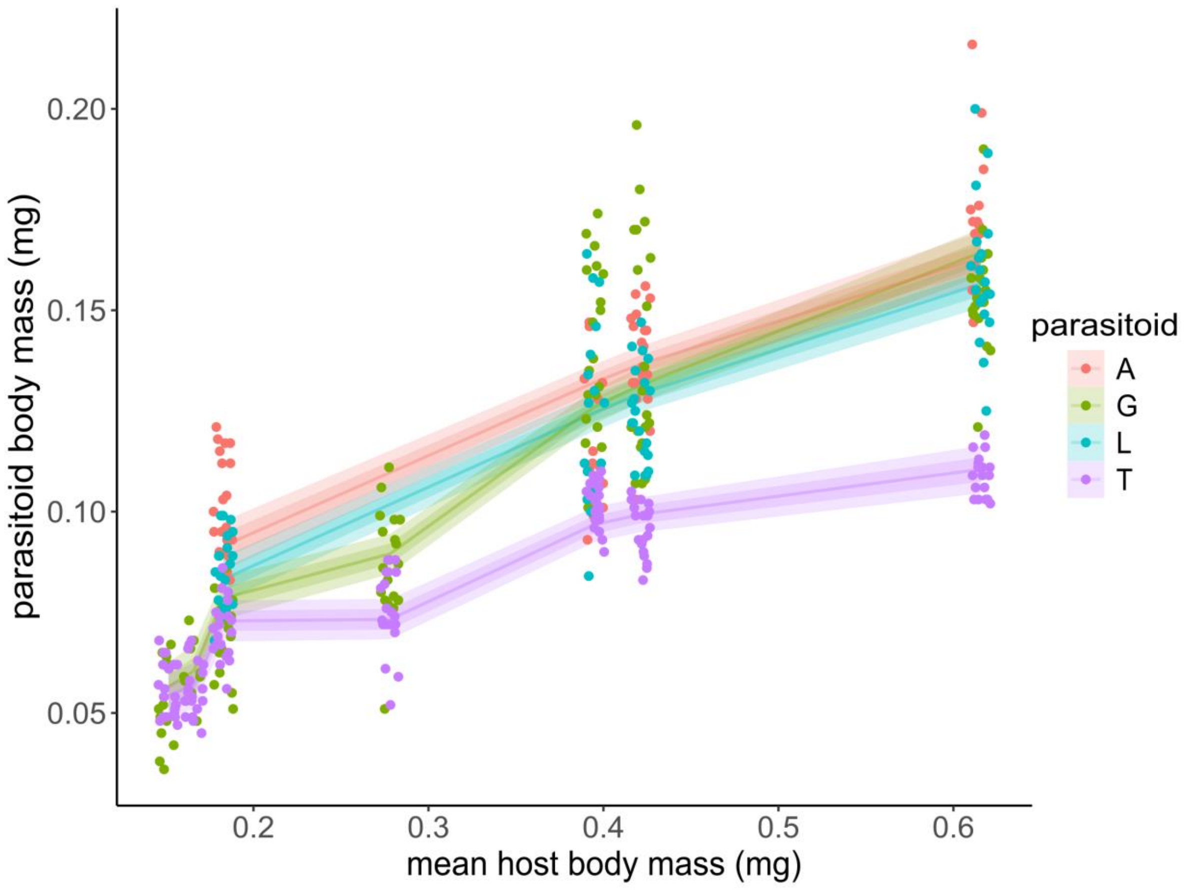

Figure S10. Correlation of parasitoid dry body mass with mean host dry body mass of the control group not subjected to infection at ambient temperature (24°C). There is a positive overall correlation with regression coefficient 0.19 and 95% confidence interval of 0.03–0.34. For more details on host body mass see Fig. S8. Parasitoid body mass was weighted in the same way from 20 individuals, if available. The minimum number of weighted parasitoids on a given host species was 10. Parasitoid species codes: A: *Asobara* sp., L: *Leptopilina* sp., G: *Ganaspis* sp., T: *Trichopria* sp. A multilevel model was fit with parasitoid body mass as the response variable with a normal distribution, and predictors included mean fly body mass as a fixed parameter, host as a grouping variable and parasitoid as a grouping variable with fly body mass as a group-level variable. See Table S12 for Bayesian model summary.

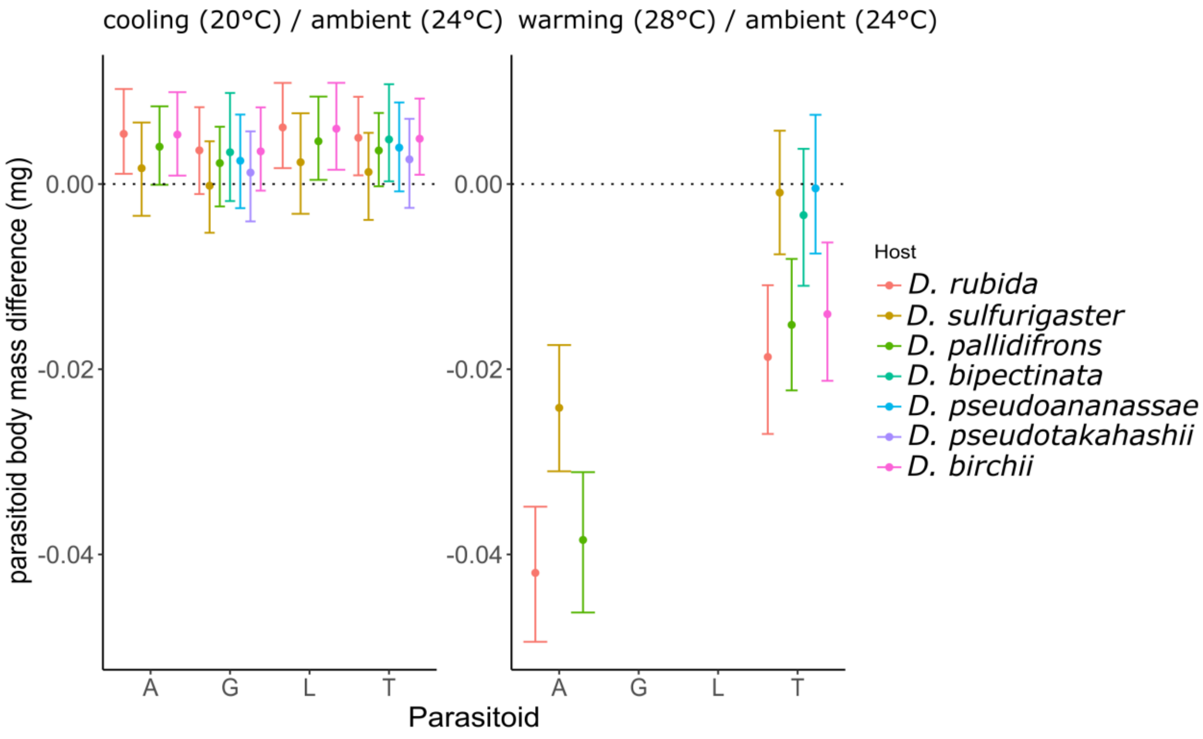

Figure S11. Thermal effect of cooling and warming on parasitoid body mass for each host – parasitoid combination where enough parasitoids hatched so that they could be weighted. Values below the dotted line (lower than 0) mean higher body mass at ambient temperature, values above the line mean higher body mass in cooling or warming, respectively. When the 95% confidence interval shown does not include 0 the difference can be considered significant. Some confidence intervals are large as a result of small number of individuals hatching at the given temperature. Parasitoid species codes: A: *Asobara* sp., L: *Leptopilina* sp., G: *Ganaspis* sp., T: *Trichopria* sp. Here a model similar to the one presented in Fig. S8 was fit, just with parasitoid body mass as the response variable instead of host body mass. All predictors were the same. See Table S13 for Bayesian model summary.

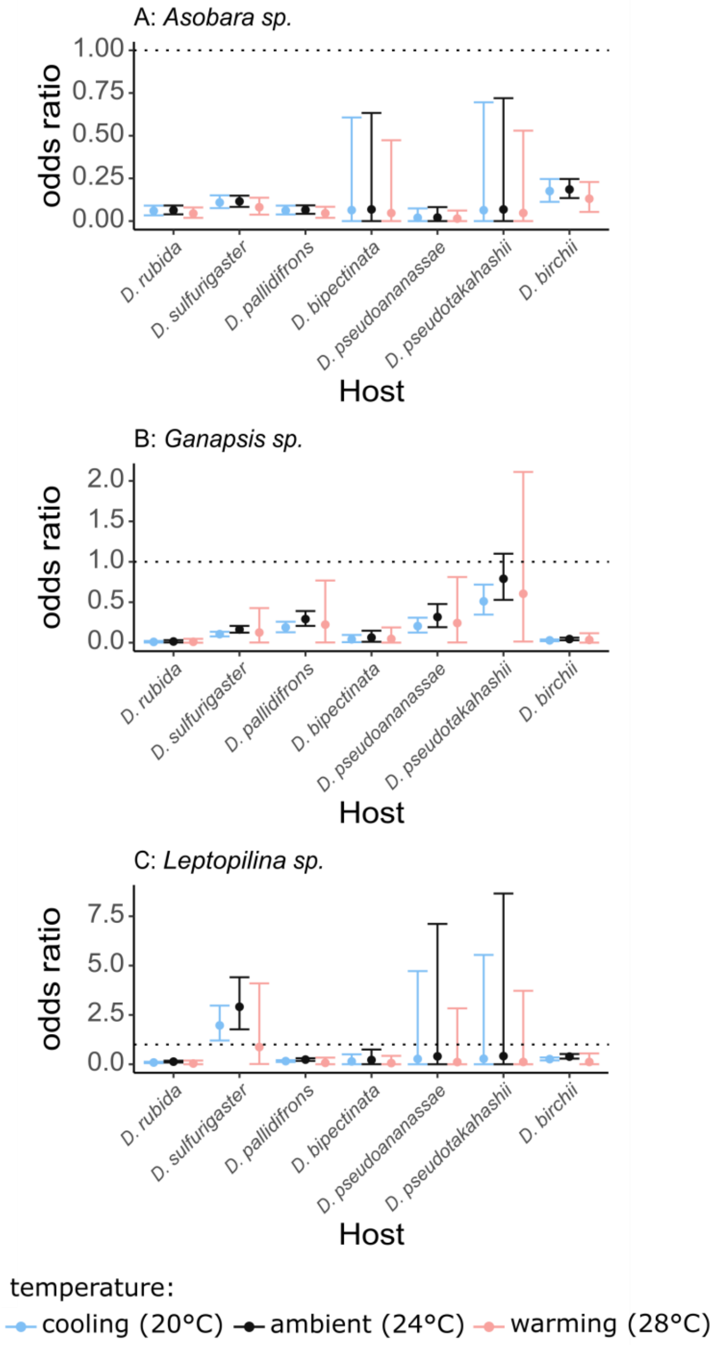

Figure S12. Change in parasitism success of the three larval parasitoid species under multiple infection from all three larval parasitoids simultaneously, compared to single infection for each *Drosophila* host at cooling, ambient and warming temperatures. The response variable represented here is the odds ratio between the parasitism success under multiple and single infection, and its credible interval, estimated by a Bayesian regression model as described in Tables S14-S16. Points below the dotted line signify competition and above the dotted line signify facilitation. In each case there were three wasps placed in each vial, in single infections they belonged to the same species, and in multiple infections they were one each from *Asobara* sp., *Ganaspis* sp., and *Leptopilina* sp. larval parasitoids. Remaining methods were the same as for the single infections.

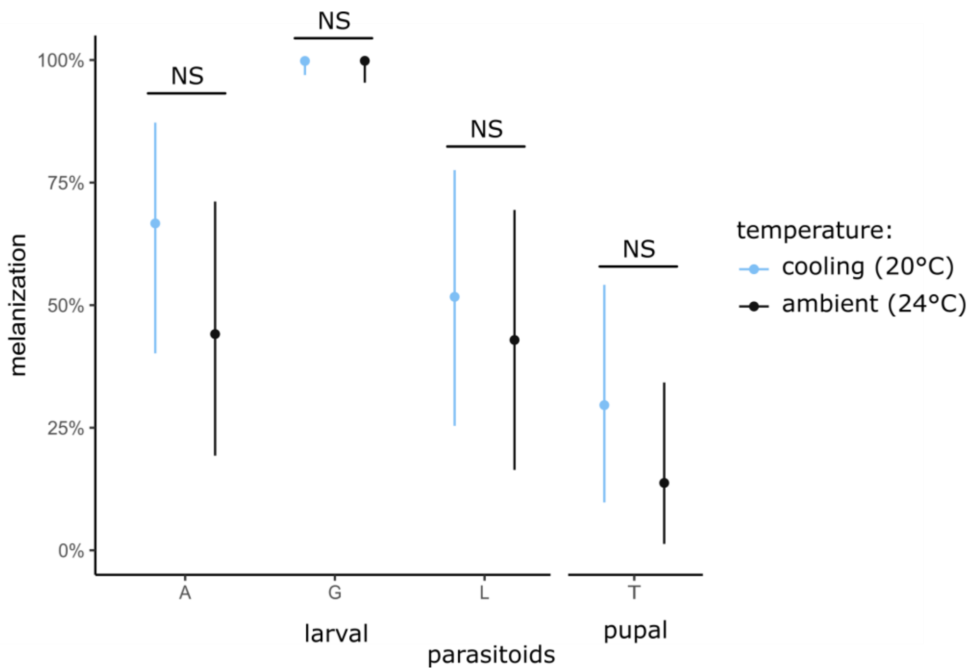

Figure S13. Melanisation in *Drosophila pseudotakahashii*. We screened all species in the experiment for melanisation, but only found melanisation in *D. pseudotakahashii*. It was not possible to score melanisation in the warming (28°C) treatment because no *D. pseudotakahashii* adults hatched. Melanised capsule of a parasitoid egg can be tricky to tell from other melanisation in *D. pseudotakahashii*. We therefore blindly scored melanisation in all parasitoid treatments, including the controls unexposed to parasitoids, to account for background melanisation not caused by parasitoids. In the figure we present raw data of melanisation – the mean and the 95% credible interval. However, the tests comparing melanisation between temperatures and species were performed using contrasts of each wasp treatment against the control at a given temperature and therefore test differences in encapsulation rate rather than raw melanisation rate. We used Bayesian regression model for these tests, described in Tables S17 and S18. Mean melanisation in the control treatment unexposed to parasitoids was 27% at 20°C and 12% at 24°C. Melanisation in all three larval parasitoids was significantly different from control. Melanisation in *Trichopria* sp. was not significantly different from control melanisation, which is consistent with the natural history of this parasitoid developing externally on the host. Parasitoid species codes: A: *Asobara* sp., L: *Leptopilina* sp., G: *Ganaspis* sp., T: *Trichopria* sp. Contrasts for pupal parasitoid "T" come from a separate model, which is signified by interrupted x axis.

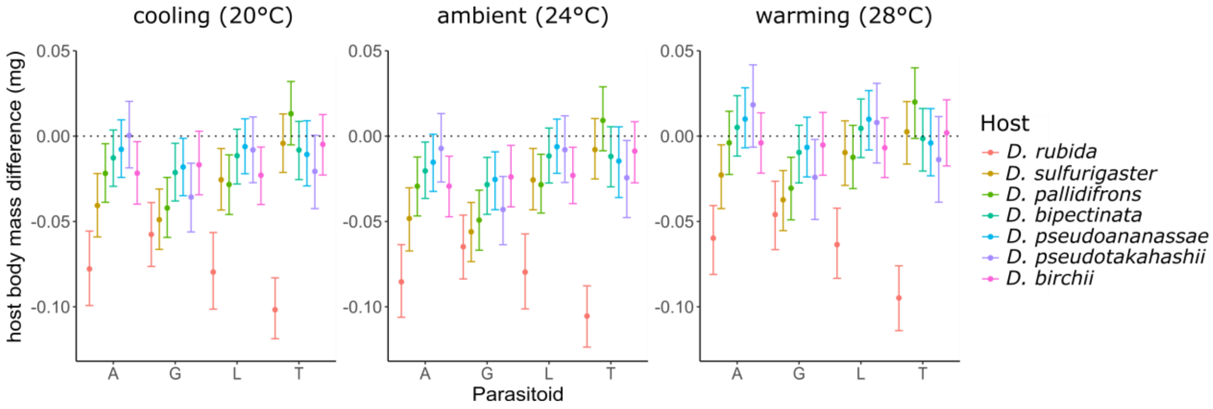

Figure S14. Contrast between body mass of *Drosophila* hosts hatching from parasitized vials vs. control vials not exposed to parasitoids. Contrast are shown for all combinations of temperature, host species and parasitoid species. Values below the dotted line (lower than 0) mean higher body mass in controls, values above the line mean higher body mass in parasitized vials. When the 95% confidence interval shown does not include 0 the difference can be considered significant. Parasitoid species codes: A: *Asobara* sp., L: *Leptopilina* sp., G: *Ganaspis* sp., T: *Trichopria* sp. Estimates here were obtained from the same model as Fig S8 and S9, as well as Table S11. Please refer to those for further details.

Table S1. Summary of the Bayesian regression model used for the analysis of parasitism success of larval parasitoids. Results from a multilevel model including temperature as a categorical fixed parameter and three grouping variables: host as a categorical variable, host accounting for phylogenetic correlations, and parasitoid. For all grouping variables, temperature was also included as a group-level variable. Models were built using the R package *brms*, with eight chains, each with 1250 iterations after the 1000 iteration warmup, totalling 10^4 posterior samples. Estimate: mean of the posterior distribution samples, SE: Standard error of the estimate, calculated as the standard deviation of the posterior samples. lower and upper CI: lower and upper 95% credible intervals, also obtained from the posterior distribution. Rhat: the potential scale reduction factor on split chains (at convergence, Rhat = 1). Bulk and tail-ESS: diagnostic of autocorrelation in the posterior samples, the effective number of posterior samples at the bulk and tails of the posterior distribution. Rhat and ESS calculated following Vehtari et al. *(2021)*.

Fixed effect Coefficients:							
	Estimate	SE	lower CI	upper CI	Rhat	Bulk_ESS	Tail_ESS
Intercept	-0.41	1.37	-3.04	2.43	1.00	4253	5449
Temp20	-0.28	1.18	-2.54	2.12	1.00	3949	4207
Temp28	-3.23	1.66	-6.4	0.31	1.00	4207	4639
Multilevel Hyperparameters							
~Host (Number of levels: 7)							
	Estimate	SE	lower CI	upper CI	Rhat	Bulk_ESS	Tail_ESS
sd(Intercept)	0.63	0.62	0.02	2.25	1.01	2261	3309
sd(Temp20)	0.83	0.48	0.1	1.99	1.00	2617	2216
sd(Temp28)	0.57	0.45	0.04	1.68	1.00	3835	3579
cor(Intercept,Temp20)	-0.02	0.5	-0.87	0.87	1.00	3241	5808
cor(Intercept,Temp28)	-0.17	0.5	-0.93	0.82	1.00	4873	6518
cor(Temp20,Temp28)	-0.23	0.47	-0.94	0.72	1.00	6805	7954
~Host Phylogenetic (Number of levels: 7)							
	Estimate	SE	lower CI	upper CI	Rhat	Bulk_ESS	Tail_ESS
sd(Intercept)	1.8	0.72	0.59	3.55	1.00	3907	2150
sd(Temp20)	1.04	0.73	0.06	2.84	1.00	2636	4341
sd(Temp28)	0.72	0.63	0.03	2.38	1.00	3907	3417
cor(Intercept,Temp20)	-0.25	0.43	-0.92	0.67	1.00	5271	6319
cor(Intercept,Temp28)	-0.29	0.45	-0.93	0.71	1.00	6467	5896
cor(Temp20,Temp28)	0.01	0.5	-0.86	0.89	1.00	7558	7720
~Parasitoid (Number of levels: 3)							
	Estimate	SE	lower CI	upper CI	Rhat	Bulk_ESS	Tail_ESS
sd(Intercept)	1.36	0.98	0.39	3.99	1.00	4599	7000
sd(Temp20)	1.03	0.87	0.29	3.19	1.00	4484	5019
sd(Temp28)	2.31	1.36	0.85	5.81	1.00	5428	6519
cor(Intercept,Temp20)	-0.12	0.45	-0.87	0.73	1.00	7210	6369
cor(Intercept,Temp28)	0.05	0.43	-0.76	0.81	1.00	7688	5872
cor(Temp20,Temp28)	-0.37	0.44	-0.96	0.6	1.00	6535	7290

Table S2. Summary of the Bayesian regression model used for the analysis of parasitism success of the pupal parasitoid. Results from a multilevel model including temperature as a categorical fixed parameter and two grouping variables: host as a categorical variable, and host accounting for phylogenetic correlations. For all grouping variables, temperature was also included as a group-level variable. Models were built using the R package *brms*, with eight chains, each with 1250 iterations after the 1000 iteration warmup, totalling 10^4 posterior samples. Estimate: mean of the posterior distribution samples, SE: Standard error of the estimate, calculated as the standard deviation of the posterior samples. lower and upper CI: lower and upper 95% credible intervals, also obtained from the posterior distribution. Rhat: the potential scale reduction factor on split chains (at convergence, Rhat = 1). Bulk and tail-ESS: diagnostic of autocorrelation in the posterior samples, the effective number of posterior samples at the bulk and tails of the posterior distribution. Rhat and ESS calculated following Vehtari et al. *(2021)*.

Fixed effect Coefficients:							
	Estimate	SE	lower CI	upper CI	Rhat	Bulk_ESS	Tail_ESS
Intercept	0.77	1.11	-1.5	3.05	1.00	5325	5381
Temp20	-0.62	0.7	-2.13	0.8	1.00	5021	4758
Temp28	-0.75	1.09	-3.17	1.42	1.00	4890	4684
Multilevel Hyperparameters							
~Host (Number of levels: 7)							
	Estimate	SE	lower CI	upper CI	Rhat	Bulk_ESS	Tail_ESS
sd(Intercept)	0.95	0.69	0.05	2.54	1.00	2460	4709
sd(Temp20)	0.63	0.41	0.04	1.63	1.00	2801	3444
sd(Temp28)	1.04	0.63	0.12	2.59	1.00	3872	3763
cor(Intercept,Temp20)	-0.37	0.48	-0.97	0.73	1.00	3342	5170
cor(Intercept,Temp28)	-0.17	0.48	-0.92	0.79	1.00	5334	6690
cor(Temp20,Temp28)	0.22	0.48	-0.76	0.92	1.00	5606	7461
~Host Phylogenetic (Number of levels: 7)							
	Estimate	SE	lower CI	upper CI	Rhat	Bulk_ESS	Tail_ESS
sd(Intercept)	1.69	0.9	0.16	3.64	1.00	2533	3619
sd(Temp20)	0.85	0.65	0.03	2.39	1.00	2654	5257
sd(Temp28)	1.27	0.98	0.05	3.66	1.00	3501	5113
cor(Intercept,Temp20)	-0.37	0.47	-0.96	0.75	1.00	3644	6376
cor(Intercept,Temp28)	-0.08	0.5	-0.89	0.85	1.00	6861	6969
cor(Temp20,Temp28)	0.16	0.51	-0.84	0.93	1.00	6249	6917

Table S3. Summary of the Bayesian regression model used for the analysis of degree of infestation of larval parasitoids. Results from a multilevel model including temperature as a categorical fixed parameter and three grouping variables: host as a categorical variable, host accounting for phylogenetic correlations, and parasitoid. For all grouping variables, temperature was also included as a group-level variable. Models were built using the R package *brms*, with eight chains, each with 1250 iterations after the 1000 iteration warmup, totalling 10^4 posterior samples. Estimate: mean of the posterior distribution samples, SE: Standard error of the estimate, calculated as the standard deviation of the posterior samples. lower and upper CI: lower and upper 95% credible intervals, also obtained from the posterior distribution. Rhat: the potential scale reduction factor on split chains (at convergence, Rhat = 1). Bulk and tail-ESS: diagnostic of autocorrelation in the posterior samples, the effective number of posterior samples at the bulk and tails of the posterior distribution. Rhat and ESS calculated following Vehtari et al. *(2021)*.

Fixed effect Coefficients:

	Estimate	SE	lower CI	upper CI	Rhat	Bulk_ESS	Tail_ESS
Intercept	0.18	1.33	-2.48	2.87	1.00	5973	6460
Temp20	0.26	1.03	-1.88	2.4	1.00	5648	5217
Temp28	-0.42	1.06	-2.41	1.54	1.00	4779	3531
Multilevel Hyperparame							
~Host (Number of levels: 7)							
	Estimate	SE	lower CI	upper CI	Rhat	Bulk_ESS	Tail_ESS
sd(Intercept)	0.87	0.68	0.04	2.55	1.00	2942	5157
sd(Temp20)	0.75	0.49	0.05	1.92	1.00	3572	3980
sd(Temp28)	0.5	0.38	0.04	1.48	1.00	3726	2845
cor(Intercept,Temp20)	-0.07	0.48	-0.89	0.83	1.00	6599	6976
cor(Intercept,Temp28)	0.03	0.49	-0.83	0.89	1.00	6117	6660
cor(Temp20,Temp28)	-0.01	0.48	-0.86	0.85	1.00	7716	8091
~Host Phylogenetic (Number of levels: 7)							
	Estimate	SE	lower CI	upper CI	Rhat	Bulk_ESS	Tail_ESS
sd(Intercept)	1.8	0.86	0.28	3.78	1.00	4214	2966
sd(Temp20)	1	0.74	0.05	2.74	1.00	3215	4825
sd(Temp28)	0.75	0.57	0.06	2.2	1.00	5211	4856
cor(Intercept,Temp20)	-0.05	0.46	-0.87	0.81	1.00	7889	6903
cor(Intercept,Temp28)	-0.24	0.45	-0.93	0.69	1.00	8502	7109
cor(Temp20,Temp28)	0.05	0.49	-0.85	0.9	1.00	7457	7478
~Parasitoid (Number of levels: 3)							
	Estimate	SE	lower CI	upper CI	Rhat	Bulk_ESS	Tail_ESS
sd(Intercept)	1.37	0.91	0.43	3.88	1.00	5529	7343
sd(Temp20)	0.85	0.74	0.21	2.96	1.00	4952	6971
sd(Temp28)	1.05	0.96	0.24	3.52	1.00	5117	5790
cor(Intercept,Temp20)	-0.36	0.46	-0.98	0.65	1.00	8436	6147
cor(Intercept,Temp28)	-0.17	0.45	-0.89	0.72	1.00	9038	6865
cor(Temp20,Temp28)	0.14	0.47	-0.77	0.89	1.00	8544	7033

Table S4. Summary of the Bayesian regression model used for the analysis of degree of infestation of the pupal parasitoid. Results from a multilevel model including temperature as a categorical fixed parameter and two grouping variables: host as a categorical variable, and host accounting for phylogenetic correlations. For all grouping variables, temperature was also included as a group-level variable. Models were built using the R package *brms*, with eight chains, each with 1250 iterations after the 1000 iteration warmup, totalling 10^4 posterior samples. Estimate: mean of the posterior distribution samples, SE: Standard error of the estimate, calculated as the standard deviation of the posterior samples. lower and upper CI: lower and upper 95% credible intervals, also obtained from the posterior distribution. Rhat: the potential scale reduction factor on split chains (at convergence, Rhat = 1). Bulk and tail-ESS: diagnostic of autocorrelation in the posterior samples, the effective number of posterior samples at the bulk and tails of the posterior distribution. Rhat and ESS calculated following Vehtari et al. *(2021)*.

Fixed effect Coefficients:							
	Estimate	SE	lower CI	upper CI	Rhat	Bulk_ESS	Tail_ESS
Intercept	1.49	0.72	-0.05	2.86	1.00	4190	5444
Temp20	-0.8	0.52	-1.83	0.32	1.00	4362	4500
Temp28	0.59	0.99	-1.49	2.56	1.00	3912	3893
Multilevel Hyperparameters							
~Host (Number of levels: 7)							
	Estimate	SE	lower CI	upper CI	Rhat	Bulk_ESS	Tail_ESS
sd(Intercept)	0.56	0.43	0.02	1.59	1.00	2564	4167
sd(Temp20)	0.4	0.29	0.02	1.1	1.00	2929	3521
sd(Temp28)	0.74	0.56	0.04	2.12	1.00	3732	4543
cor(Intercept,Temp20)	-0.27	0.5	-0.95	0.78	1.00	4543	6909
cor(Intercept,Temp28)	-0.11	0.49	-0.89	0.83	1.00	6720	7339
cor(Temp20,Temp28)	-0.13	0.49	-0.92	0.81	1.00	6638	7169
~Host Phylogenetic (Number of levels: 7)							
	Estimate	SE	lower CI	upper CI	Rhat	Bulk_ESS	Tail_ESS
sd(Intercept)	1.02	0.56	0.11	2.32	1.00	2748	3121
sd(Temp20)	0.64	0.44	0.04	1.7	1.00	2786	4225
sd(Temp28)	1.22	0.82	0.09	3.29	1.00	3855	3695
cor(Intercept,Temp20)	-0.38	0.46	-0.96	0.69	1.00	4460	5884
cor(Intercept,Temp28)	-0.19	0.46	-0.91	0.74	1.00	6454	6230
cor(Temp20,Temp28)	-0.02	0.48	-0.85	0.86	1.00	6486	7325

Table S5. Summary of the Bayesian regression model used for the analysis of *Drosophila* host developmental success without infection. Results from a multilevel model including temperature as a categorical fixed parameter and two grouping variables: host as a categorical variable and host accounting for phylogenetic correlations. For all grouping variables, temperature was also included as a group-level variable. Models were built using the R package *brms*, with eight chains, each with 1250 iterations after the 1000 iteration warmup, totalling 10^4 posterior samples. Estimate: mean of the posterior distribution samples, SE: Standard error of the estimate, calculated as the standard deviation of the posterior samples. lower and upper CI: lower and upper 95% credible intervals, also obtained from the posterior distribution. Rhat: the potential scale reduction factor on split chains (at convergence, Rhat = 1). Bulk and tail-ESS: diagnostic of autocorrelation in the posterior samples, the effective number of posterior samples at the bulk and tails of the posterior distribution. Rhat and ESS calculated following Vehtari et al. *(2021)*.

Fixed effect Coefficients:

	Esti-mate	SE	lower CI	upper CI	Rhat	Bulk_ESS	Tail_ESS
Intercept	0.75	0.74	-0.83	2.22	1.00	3277	4382
Temp20	-0.06	0.39	-0.87	0.76	1.00	2651	2563
Temp28	-1.88	1.94	-5.99	1.89	1.00	3126	3667

Multilevel Hyperparameters

~Host (Number of levels: 7)

	Esti-mate	SE	lower CI	upper CI	Rhat	Bulk_ESS	Tail_ESS
sd(Intercept)	0.76	0.45	0.06	1.8	1.00	1750	2258
sd(Temp20)	0.43	0.25	0.08	1.05	1.00	2879	1934
sd(Temp28)	1.72	1.21	0.11	4.51	1.00	1966	3159
cor(Intercept,Temp20)	-0.25	0.43	-0.89	0.68	1.00	2763	4101
cor(Intercept,Temp28)	0.16	0.47	-0.8	0.9	1.00	3049	4529
cor(Temp20,Temp28)	0.12	0.44	-0.74	0.88	1.00	4827	6264

~Host Phylogenetic (Number of levels: 7)

	Esti-mate	SE	lower CI	upper CI	Rhat	Bulk_ESS	Tail_ESS
sd(Intercept)	0.94	0.69	0.04	2.56	1.00	1568	3601
sd(Temp20)	0.41	0.4	0.01	1.48	1.00	2032	3295
sd(Temp28)	2.69	1.68	0.16	6.5	1.00	1779	2493
cor(Intercept,Temp20)	-0.13	0.51	-0.93	0.85	1.00	3847	5006
cor(Intercept,Temp28)	0.17	0.47	-0.8	0.91	1.00	3106	4228
cor(Temp20,Temp28)	-0.02	0.49	-0.88	0.86	1.00	3924	6012

Table S6. *Model without host phylogeny, analogous to Table S1.* Summary of the Bayesian regression model used for the analysis of parasitism success of larval parasitoids. Results from a multilevel model including temperature as a categorical fixed parameter and two grouping variables: host as a categorical variable and parasitoid. For all grouping variables, temperature was also included as a group-level variable. Models were built using the R package *brms*, with eight chains, each with 1250 iterations after the 1000 iteration warmup, totalling 10^4 posterior samples. Estimate: mean of the posterior distribution samples, SE: Standard error of the estimate, calculated as the standard deviation of the posterior samples. lower and upper CI: lower and upper 95% credible intervals, also obtained from the posterior distribution. Rhat: the potential scale reduction factor on split chains (at convergence, Rhat = 1). Bulk and tail-ESS: diagnostic of autocorrelation in the posterior samples, the effective number of posterior samples at the bulk and tails of the posterior distribution. Rhat and ESS calculated following Vehtari et al. *(2021)*.

Fixed effect Coefficients:							
	Estimate	SE	lower CI	upper CI	Rhat	Bulk_ESS	Tail_ESS
Intercept	-0.87	1.15	-2.93	1.63	1.00	3424	4791
Temp20	-0.18	0.86	-1.83	1.6	1.00	3335	3792
Temp28	-3.1	1.5	-5.94	0.21	1.00	4593	4262
Multilevel Hyperparameters							
~Host (Number of levels: 7)							
	Estimate	SE	lower CI	upper CI	Rhat	Bulk_ESS	Tail_ESS
sd(Intercept)	1.99	0.6	1.17	3.41	1.00	4273	6348
sd(Temp20)	1.1	0.39	0.6	2.11	1.00	3932	5912
sd(Temp28)	0.65	0.36	0.15	1.5	1.00	3951	3716
cor(Intercept,Temp20)	-0.44	0.29	-0.86	0.24	1.00	5273	6229
cor(Intercept,Temp28)	-0.51	0.33	-0.94	0.29	1.00	7565	7155
cor(Temp20,Temp28)	-0.1	0.38	-0.77	0.65	1.00	9252	7801
~Parasitoid (Number of levels: 3)							
	Estimate	SE	lower CI	upper CI	Rhat	Bulk_ESS	Tail_ESS
sd(Intercept)	1.37	0.98	0.38	3.97	1.00	5573	6808
sd(Temp20)	1.01	0.82	0.29	3.21	1.00	4438	4391
sd(Temp28)	2.25	1.28	0.83	5.63	1.00	5147	5886
cor(Intercept,Temp20)	-0.13	0.45	-0.86	0.75	1.00	7877	6561
cor(Intercept,Temp28)	0.04	0.43	-0.76	0.8	1.00	9411	7023
cor(Temp20,Temp28)	-0.37	0.43	-0.96	0.59	1.00	5327	7130

Table S7. *Model without host phylogeny, analogous to Table S2.* Summary of the Bayesian regression model used for the analysis of parasitism success of the pupal parasitoid. Results from a multilevel model including temperature as a categorical fixed parameter and host as a grouping variable. For all grouping variables, temperature was also included as a group-level variable. Models were built using the R package brms, with eight chains, each with 1250 iterations after the 1000 iteration warmup, totalling 10^4 posterior samples. Estimate: mean of the posterior distribution samples, SE: Standard error of the estimate, calculated as the standard deviation of the posterior samples. lower and upper CI: lower and upper 95% credible intervals, also obtained from the posterior distribution. Rhat: the potential scale reduction factor on split chains (at convergence, Rhat = 1). Bulk and tail-ESS: diagnostic of autocorrelation in the posterior samples, the effective number of posterior samples at the bulk and tails of the posterior distribution. Rhat and ESS calculated following Vehtari et al. *(2021)*.

Fixed effect Coefficients:

	Estimate	SE	lower CI	upper CI	Rhat	Bulk_ESS	Tail_ESS
Intercept	0.83	0.68	-0.54	2.19	1.00	3350	4539
Temp20	-0.62	0.33	-1.29	0.01	1.00	3827	4350
Temp28	-0.86	0.62	-2.15	0.33	1.00	4494	4924

Multilevel Hyperparameters

~Host (Number of levels: 7)

	Estimate	SE	lower CI	upper CI	Rhat	Bulk_ESS	Tail_ESS
sd(Intercept)	1.74	0.57	0.98	3.15	1.00	4506	5709
sd(Temp20)	0.79	0.3	0.37	1.53	1.00	4021	6036
sd(Temp28)	1.34	0.57	0.64	2.8	1.00	4604	5265
cor(Intercept,Temp20)	-0.64	0.26	-0.95	0.02	1.00	7283	6994
cor(Intercept,Temp28)	-0.22	0.4	-0.84	0.61	1.00	6883	6790
cor(Temp20,Temp28)	0.47	0.37	-0.4	0.94	1.00	6171	7210

Table S8. *Model without host phylogeny, analogous to Table S3.* Summary of the Bayesian regression model used for the analysis of degree of infestation of larval parasitoids. Results from a multilevel model including temperature as a categorical fixed parameter and two grouping variables: host and parasitoid. For all grouping variables, temperature was also included as a group-level variable. Models were built using the R package brms, with eight chains, each with 1250 iterations after the 1000 iteration warmup, totalling 10^4 posterior samples. Estimate: mean of the posterior distribution samples, SE: Standard error of the estimate, calculated as the standard deviation of the posterior samples. lower and upper CI: lower and upper 95% credible intervals, also obtained from the posterior distribution. Rhat: the potential scale reduction factor on split chains (at convergence, Rhat = 1). Bulk and tail-ESS: diagnostic of autocorrelation in the posterior samples, the effective number of posterior samples at the bulk and tails of the posterior distribution. Rhat and ESS calculated following Vehtari et al. *(2021)*.

Fixed effect Coefficients:							
	Estimate	SE	lower CI	upper CI	Rhat	Bulk_ESS	Tail_ESS
Intercept	-0.07	1.03	-2.12	1.98	1.00	4815	6017
Temp20	0.32	0.8	-1.18	1.85	1.00	4618	4807
Temp28	-0.33	0.78	-1.93	1.21	1.00	5803	5140
Multilevel Hyperparame							
~Host (Number of levels: 7)							
	Estimate	SE	lower CI	upper CI	Rhat	Bulk_ESS	Tail_ESS
sd(Intercept)	1.95	0.64	1.1	3.56	1.00	4509	6363
sd(Temp20)	1.01	0.4	0.51	2.07	1.00	5917	7420
sd(Temp28)	0.68	0.3	0.34	1.46	1.00	5188	5562
cor(Intercept,Temp20)	-0.11	0.33	-0.7	0.55	1.00	6334	6499
cor(Intercept,Temp28)	-0.48	0.32	-0.91	0.26	1.00	7050	6417
cor(Temp20,Temp28)	0.11	0.37	-0.61	0.75	1.00	8833	7416
~Parasitoid (Number of levels: 3)							
	Estimate	SE	lower CI	upper CI	Rhat	Bulk_ESS	Tail_ESS
sd(Intercept)	1.34	0.87	0.44	3.6	1.00	6177	6766
sd(Temp20)	0.88	0.83	0.21	3.11	1.00	4573	4668
sd(Temp28)	1.03	0.89	0.24	3.37	1.00	6225	5737
cor(Intercept,Temp20)	-0.36	0.47	-0.97	0.67	1.00	7997	6147
cor(Intercept,Temp28)	-0.16	0.45	-0.88	0.73	1.00	11036	7434
cor(Temp20,Temp28)	0.13	0.47	-0.77	0.89	1.00	8474	7070

Table S9. *Model without host phylogeny, analogous to Table S4.* Summary of the Bayesian regression model used for the analysis of degree of infestation of the pupal parasitoid. Results from a multilevel model including temperature as a categorical fixed parameter and host as a grouping variables. For all grouping variables, temperature was also included as a group-level variable. Models were built using the R package *brms*, with eight chains, each with 1250 iterations after the 1000 iteration warmup, totalling 10^4 posterior samples. Estimate: mean of the posterior distribution samples, SE: Standard error of the estimate, calculated as the standard deviation of the posterior samples. lower and upper CI: lower and upper 95% credible intervals, also obtained from the posterior distribution. Rhat: the potential scale reduction factor on split chains (at convergence, Rhat = 1). Bulk and tail-ESS: diagnostic of autocorrelation in the posterior samples, the effective number of posterior samples at the bulk and tails of the posterior distribution. Rhat and ESS calculated following Vehtari et al. *(2021)*.

Fixed effect Coefficients:

	Estimate	SE	lower CI	upper CI	Rhat	Bulk_ESS	Tail_ESS
Intercept	1.71	0.41	0.88	2.53	1.00	3011	4306
Temp20	-0.87	0.25	-1.37	-0.37	1.00	3494	4628
Temp28	0.56	0.52	-0.51	1.62	1.00	4793	5094

Multilevel Hyperparameters

~Host (Number of levels: 7)

	Estimate	SE	lower CI	upper CI	Rhat	Bulk_ESS	Tail_ESS
sd(Intercept)	1.01	0.32	0.57	1.8	1.00	4313	5566
sd(Temp20)	0.58	0.22	0.28	1.12	1.00	4428	5940
sd(Temp28)	1.19	0.5	0.55	2.43	1.00	4687	5953
cor(Intercept,Temp20)	-0.71	0.23	-0.97	-0.08	1.00	5800	6892
cor(Intercept,Temp28)	-0.43	0.3	-0.87	0.27	1.00	5639	6579
cor(Temp20,Temp28)	-0.01	0.37	-0.7	0.68	1.00	7166	7066

Table S10. *Model without host phylogeny, analogous to Table S5.* Summary of the Bayesian regression model used for the analysis of *Drosophila* host developmental success without infection. Results from a multilevel model including temperature as a categorical fixed parameter and host as grouping variable. For all grouping variables, temperature was also included as a group-level variable. Models were built using the R package *brms*, with eight chains, each with 1250 iterations after the 1000 iteration warmup, totalling 10^4 posterior samples. Estimate: mean of the posterior distribution samples, SE: Standard error of the estimate, calculated as the standard deviation of the posterior samples. lower and upper CI: lower and upper 95% credible intervals, also obtained from the posterior distribution. Rhat: the potential scale reduction factor on split chains (at convergence, Rhat = 1). Bulk and tail-ESS: diagnostic of autocorrelation in the posterior samples, the effective number of posterior samples at the bulk and tails of the posterior distribution. Rhat and ESS calculated following Vehtari et al. *(2021)*.

Fixed effect Coefficients:

	Esti-mate	SE	lower CI	upper CI	Rhat	Bulk_ESS	Tail_ESS
Intercept	0.82	0.37	0.09	1.57	1.00	3671	5221
Temp20	-0.08	0.2	-0.47	0.31	1.00	4953	5065
Temp28	-1.65	1.1	-3.97	0.46	1.00	4506	5326

Multilevel Hyperparameters

~Host (Number of levels: 7)

	Esti-mate	SE	lower CI	upper CI	Rhat	Bulk_ESS	Tail_ESS
sd(Intercept)	0.96	0.34	0.53	1.82	1.00	4294	6138
sd(Temp20)	0.44	0.2	0.19	0.92	1.00	4134	5843
sd(Temp28)	2.78	1.07	1.39	5.39	1.00	4941	6495
cor(Intercept,Temp20)	-0.39	0.33	-0.88	0.36	1.00	7362	7437
cor(Intercept,Temp28)	0.33	0.3	-0.34	0.81	1.00	6972	6498
cor(Temp20,Temp28)	0.02	0.34	-0.62	0.66	1.00	6260	6849

Table S11. Summary of the Bayesian regression model used for the analysis of fly adult dry body mass. Results from a multilevel model including temperature as a categorical fixed parameter and two grouping variables: parasitoid, with temperature as a group-level variable, and host with both parasitoid and temperature and group-level variables. Models were built using the R package brms, with eight chains, each with 1250 iterations after the 1000 iteration warmup, totalling 10^4 posterior samples. Estimate: mean of the posterior distribution samples, SE: Standard error of the estimate, calculated as the standard deviation of the posterior samples. lower and upper CI: lower and upper 95% credible intervals, also obtained from the posterior distribution. Rhat: the potential scale reduction factor on split chains (at convergence, Rhat = 1). Bulk and tail-ESS: diagnostic of autocorrelation in the posterior samples, the effective number of posterior samples at the bulk and tails of the posterior distribution. Rhat and ESS calculated following Vehtari et al. *(2021)*.

Fixed effect Coefficients:

	Estimate	SE	lower CI	upper CI	Rhat	Bulk_ESS	Tail_ESS
Intercept	2.620	0.680	1.230	3.97	1.00	2238	3954
Temp24	0.020	0.090	-0.150	0.18	1.00	4671	5374
Temp28	-0.46	0.3	-1.06	0.16	1.00	3223	4298

~Host (Number of levels: 7)

	Estimate	SE	lower CI	upper CI	Rhat	Bulk_ESS	Tail_ESS
sd(Intercept)	1.71	5.30E-01	1	3.04	1.00	3608	5245
sd(Temp24)	0.14	0.06	0.06	0.3	1.00	4657	5637
sd(Temp28)	0.67	0.29	0.35	1.4	1.00	4468	4748
sd(ParasitoidC)	0.34	0.14	0.17	0.7	1.00	3828	6199
sd(ParasitoidT)	0.38	0.16	0.19	0.78	1.00	6184	6750
sd(ParasitoidG)	0.27	0.12	0.11	0.55	1.00	5320	6611
sd(ParasitoidL)	0.14	0.08	0.02	0.33	1.00	4535	3854
cor(Intercept,Temp24)	-0.38	0.29	-0.84	0.24	1.00	8102	7692
cor(Intercept,Temp28)	-0.39	0.27	-0.82	0.22	1.00	6823	6952
cor(Temp24,Temp28)	0.37	0.3	-0.29	0.85	1.00	7324	7375
cor(Intercept,ParasitoidC)	0.36	0.28	-0.24	0.81	1.00	5721	6174
cor(Temp24,ParasitoidC)	-0.27	0.31	-0.8	0.38	1.00	5889	6587
cor(Temp28,ParasitoidC)	-0.18	0.3	-0.7	0.44	1.00	6449	7721
cor(Intercept,ParasitoidT)	-0.04	0.27	-0.55	0.49	1.00	9410	7369
cor(Temp24,ParasitoidT)	-0.13	0.3	-0.67	0.47	1.00	7133	7654
cor(Temp28,ParasitoidT)	-0.03	0.29	-0.58	0.54	1.00	7118	7858
cor(ParasitoidC,ParasitoidT)	-0.05	0.29	-0.59	0.52	1.00	8333	8148
cor(Intercept,ParasitoidG)	-0.02	0.28	-0.56	0.53	1.00	8105	7291
cor(Temp24,ParasitoidG)	-0.15	0.31	-0.69	0.47	1.00	7213	7959
cor(Temp28,ParasitoidG)	-0.05	0.31	-0.63	0.56	1.00	7211	7812
cor(ParasitoidC,ParasitoidG)	0.33	0.3	-0.31	0.81	1.00	5624	8444
cor(ParasitoidT,ParasitoidG)	-0.05	0.3	-0.6	0.53	1.00	9252	8445
cor(Intercept,ParasitoidL)	0	0.3	-0.56	0.57	1.00	12214	7938
cor(Temp24,ParasitoidL)	0.01	0.32	-0.6	0.61	1.00	9250	8106
cor(Temp28,ParasitoidL)	0.19	0.31	-0.46	0.74	1.00	8925	8197

cor(ParasitoidC,ParasitoidL)	0.15	0.32	-0.49	0.71	1.00	9840	8905
cor(ParasitoidT,ParasitoidL)	0.19	0.32	-0.46	0.73	1.00	9246	8561
cor(ParasitoidG,ParasitoidL)	0.11	0.32	-0.53	0.69	1.00	8714	8555

~Parasitoid (Number of levels: 5)

	Estimate	SE	lower CI	upper CI	Rhat	Bulk_ESS	Tail_ESS
sd(Intercept)	0.17	0.15	0.01	0.55	1.00	2365	3752
sd(Temp24)	0.09	0.09	0	0.33	1.00	4027	4420
sd(Temp28)	0.14	0.11	0.02	0.42	1.00	4254	4085
cor(Intercept,Temp24)	0.13	0.48	-0.81	0.92	1.00	7485	5901
cor(Intercept,Temp28)	-0.31	0.47	-0.95	0.71	1.00	5620	6355
cor(Temp24,Temp28)	-0.07	0.48	-0.89	0.81	1.00	7516	7868

Table S12. Summary of the Bayesian regression model used for the analysis of dependence of parasitoid adult dry body mass on fly adult dry body mass at ambient temperature. Results from a multilevel model including mean fly body mass as a fixed parameter, host as a grouping variable and parasitoid as a grouping variable with fly body mass as a group-level variable. Models were built using the R package brms, with eight chains, each with 1250 iterations after the 1000 iteration warmup, totalling 10^4 posterior samples. Estimate: mean of the posterior distribution samples, SE: Standard error of the estimate, calculated as the standard deviation of the posterior samples. lower and upper CI: lower and upper 95% credible intervals, also obtained from the posterior distribution. Rhat: the potential scale reduction factor on split chains (at convergence, Rhat = 1). Bulk and tail-ESS: diagnostic of autocorrelation in the posterior samples, the effective number of posterior samples at the bulk and tails of the posterior distribution. Rhat and ESS calculated following Vehtari et al. *(2021)*.

Fixed effect Coefficients:

	Estimate	SE	lower CI	upper CI	Rhat	Bulk_ESS	Tail_ESS
Intercept	0.38	0.17	0.03	0.71	1.00	4951	5432
fly_body mass	0.19	0.08	0.03	0.34	1.00	2840	2124

~Host (Number of levels: 7)

	Estimate	SE	lower CI	upper CI	Rhat	Bulk_ESS	Tail_ESS
sd(Intercept)	0.11	0.06	0.05	0.25	1.00	3041	4535

~Parasitoid (Number of levels: 4)

	Estimate	SE	lower CI	upper CI	Rhat	Bulk_ESS	Tail_ESS
sd(Intercept)	0.21	0.21	0.05	0.77	1.00	3500	4713
sd(fly_body mass)	0.11	0.11	0.03	0.39	1.00	2635	2225
cor(Intercept,fly_body mass)	-0.26	0.49	-0.95	0.78	1.00	3848	5356

Table S13. Summary of the Bayesian regression model used for the analysis of parasitoid adult dry body mass. Results from a multilevel model including temperature as a categorical fixed parameter and two grouping variables: parasitoid, with Temperature as a group-level variable, and host with both parasitoid and temperature and group-level variables. Models were built using the R package brms, with eight chains, each with 1250 iterations after the 1000 iteration warmup, totalling 10^4 posterior samples. Estimate: mean of the posterior distribution samples, SE: Standard error of the estimate, calculated as the standard deviation of the posterior samples. lower and upper CI: lower and upper 95% credible intervals, also obtained from the posterior distribution. Rhat: the potential scale reduction factor on split chains (at convergence, Rhat = 1). Bulk and tail-ESS: diagnostic of autocorrelation in the posterior samples, the effective number of posterior samples at the bulk and tails of the posterior distribution. Rhat and ESS calculated following Vehtari et al. *(2021)*.

Fixed effect Coefficients:

	Estimate	SE	lower CI	upper CI	Rhat	Bulk_ESS	Tail_ESS
Intercept	1.000	0.270	0.510	1.54	1.00	3847	4745
Temp24	-0.040	0.030	-0.100	0.02	1.00	5925	5094
Temp28	-0.24	0.93	-2.21	1.84	1.00	4443	3757

~Host (Number of levels: 7)

	Estimate	SE	lower CI	upper CI	Rhat	Bulk_ESS	Tail_ESS
sd(Intercept)	0.41	1.60E-01	0.21	0.82	1.00	3693	4530
sd(Temp24)	0.03	0.02	0	0.09	1.00	4511	5130
sd(Temp28)	0.13	0.07	0.06	0.3	1.00	4991	5691
sd(ParasitoidT)	0.2	0.1	0.09	0.44	1.00	3480	4534
sd(ParasitoidG)	0.24	0.1	0.11	0.49	1.00	5317	6654
sd(ParasitoidL)	0.12	0.11	0.03	0.4	1.00	4926	5590
cor(Intercept,Temp24)	0	0.34	-0.65	0.63	1.00	11700	6667
cor(Intercept,Temp28)	-0.26	0.3	-0.76	0.35	1.00	8977	7807
cor(Temp24,Temp28)	0.19	0.36	-0.54	0.8	1.00	6177	7139
cor(Intercept,ParasitoidT)	-0.55	0.28	-0.93	0.12	1.00	5492	6203
cor(Temp24,ParasitoidT)	0.14	0.35	-0.56	0.76	1.00	7234	7372
cor(Temp28,ParasitoidT)	0.29	0.32	-0.4	0.82	1.00	6784	7974
cor(Intercept,ParasitoidG)	0.24	0.31	-0.41	0.75	1.00	6399	6605
cor(Temp24,ParasitoidG)	0.19	0.35	-0.53	0.79	1.00	6062	6673
cor(Temp28,ParasitoidG)	0.07	0.33	-0.54	0.67	1.00	8066	8225
cor(ParasitoidT,ParasitoidG)	0.15	0.31	-0.46	0.7	1.00	7964	8292
cor(Intercept,ParasitoidL)	-0.05	0.33	-0.67	0.59	1.00	11008	7419
cor(Temp24,ParasitoidL)	0.17	0.37	-0.58	0.8	1.00	8107	8059
cor(Temp28,ParasitoidL)	0.27	0.35	-0.46	0.85	1.00	9209	7582
cor(ParasitoidT,ParasitoidL)	0.18	0.33	-0.5	0.76	1.00	9573	9020
cor(ParasitoidG,ParasitoidL)	0.19	0.34	-0.5	0.77	1.00	8397	8653

~Parasitoid (Number of levels: 4)

	Estimate	SE	lower CI	upper CI	Rhat	Bulk_ESS	Tail_ESS

sd(Intercept)	0.3	0.31	0.02	1.1	1.00	3095	3382
sd(Temp24)	0.04	0.05	0	0.16	1.00	3396	4959
sd(Temp28)	1.04	1.15	0.1	4.2	1.00	4121	5192
cor(Intercept,Temp24)	-0.07	0.5	-0.9	0.86	1.00	11539	6916
cor(Intercept,Temp28)	-0.14	0.46	-0.9	0.76	1.00	10282	7477
cor(Temp24,Temp28)	0.01	0.51	-0.89	0.9	1.00	9513	8004

Table S14. Summary of the Bayesian regression model used for the analysis of parasitism success of *Asobara sp*. alone or in multiple infection of different hosts. Results from a multilevel model including temperature, infection type and their interaction as categorical fixed parameters and two grouping variables: host as a categorical variable with temperature and infection type as group-level variables, and host accounting for phylogenetic correlations with temperature included as a group-level variable. Models were built using the R package *brms*, with eight chains, each with 1250 iterations after the 1000 iteration warmup, totalling 10^4 posterior samples. Estimate: mean of the posterior distribution samples, SE: Standard error of the estimate, calculated as the standard deviation of the posterior samples. lower and upper CI: lower and upper 95% credible intervals, also obtained from the posterior distribution. Rhat: the potential scale reduction factor on split chains (at convergence, Rhat = 1). Bulk and tail-ESS: diagnostic of autocorrelation in the posterior samples, the effective number of posterior samples at the bulk and tails of the posterior distribution. Rhat and ESS calculated following Vehtari et al. *(2021)*.

Fixed effect Coefficients:

	Estimate	SE	lower CI	upper CI	Rhat	Bulk_ESS	Tail_ESS
Intercept	-1.14	2.1	-5.1	3.24	1.00	5027	7007
Temp24	0.13	2.16	-4.21	4.57	1.00	4496	4606
Temp28	-1.61	1.85	-5.45	2.09	1.00	4788	4514
Multiple	-2.74	0.74	-4.49	-1.45	1.00	4912	3851
Temp24:Multiple	0.06	0.18	-0.28	0.4	1.00	15021	8064
Temp28:Multiple	-0.3	0.34	-0.99	0.34	1.00	16171	8171

Multilevel Hyperparameters

~Host (Number of levels: 7)

	Estimate	SE	lower CI	upper CI	Rhat	Bulk_ESS	Tail_ESS
sd(Intercept)	2.94	1.64	0.49	6.87	1.00	3353	3069
sd(Temp24)	1.61	1.33	0.07	4.91	1.00	2797	5671
sd(Temp28)	1.27	1.1	0.05	4.08	1.00	3639	5186
sd(Multiple)	1.31	0.81	0.4	3.43	1.00	4600	5990
cor(Intercept,Temp24)	0.02	0.46	-0.81	0.83	1.00	8256	6909
cor(Intercept,Temp28)	-0.07	0.45	-0.85	0.79	1.00	9591	7017
cor(Temp24,Temp28)	0.18	0.45	-0.73	0.9	1.00	5465	7447
cor(Intercept,Multiple)	-0.06	0.45	-0.83	0.79	1.00	4895	6237
cor(Temp24,Multiple)	0.26	0.43	-0.67	0.91	1.00	4366	5780
cor(Temp28,Multiple)	0.14	0.44	-0.74	0.85	1.00	5617	7637

~Host Phylogenetic (Number of levels: 7)

	Estimate	SE	lower CI	upper CI	Rhat	Bulk_ESS	Tail_ESS
sd(Intercept)	2.8	2.01	0.16	7.78	1.00	2051	3379
sd(Temp24)	2.81	1.79	0.21	7	1.00	2708	2597
sd(Temp28)	2.23	1.5	0.16	5.93	1.00	3550	3212
cor(Intercept,Temp24)	0.24	0.46	-0.72	0.93	1.00	4313	5979
cor(Intercept,Temp28)	0.18	0.48	-0.78	0.92	1.00	4893	6301
cor(Temp24,Temp28)	0.41	0.44	-0.64	0.96	1.00	4185	5963

Table S15. Summary of the Bayesian regression model used for the analysis of parasitism success of *Ganapsis sp*. alone or in multiple infection of different hosts. Results from a multilevel model including temperature, infection type and their interaction as categorical fixed parameters and two grouping variables: host as a categorical variable with temperature and infection type as group-level variables, and host accounting for phylogenetic correlations with temperature included as a group-level variable. Models were built using the R package *brms*, with eight chains, each with 1250 iterations after the 1000 iteration warmup, totalling 10^4 posterior samples. Estimate: mean of the posterior distribution samples, SE: Standard error of the estimate, calculated as the standard deviation of the posterior samples. lower and upper CI: lower and upper 95% credible intervals, also obtained from the posterior distribution. Rhat: the potential scale reduction factor on split chains (at convergence, Rhat = 1). Bulk and tail-ESS: diagnostic of autocorrelation in the posterior samples, the effective number of posterior samples at the bulk and tails of the posterior distribution. Rhat and ESS calculated following Vehtari et al. *(2021)*.

Fixed effect Coefficients:

	Estimate	SE	lower CI	upper CI	Rhat	Bulk_ESS	Tail_ESS
Intercept	-0.19	1.13	-2.2	2.39	1.00	5968	6172
Temp24	0.33	0.79	-1.21	2.01	1.00	5536	5196
Temp28	-4.63	2.26	-9.26	0.06	1.00	5809	5252
Multiple	-2.44	0.73	-3.91	-0.96	1.00	6476	5575
Temp24:Multiple	0.44	0.14	0.16	0.71	1.00	19201	7513
Temp28:Multiple	0.09	0.89	-1.94	1.6	1.00	14223	5714

Multilevel Hyperparameters

~Host (Number of levels: 7)

	Estimate	SE	lower CI	upper CI	Rhat	Bulk_ESS	Tail_ESS
sd(Intercept)	1.04	0.63	0.08	2.53	1	3139	3259
sd(Temp24)	0.55	0.45	0.02	1.63	1	3117	5811
sd(Temp28)	1.58	1.32	0.07	4.89	1	4826	5606
sd(Multiple)	1.79	0.65	0.94	3.33	1	7103	7456
cor(Intercept,Temp24)	-0.05	0.43	-0.82	0.77	1	8673	6903
cor(Intercept,Temp28)	-0.14	0.43	-0.87	0.72	1	8712	7277
cor(Temp24,Temp28)	-0.02	0.46	-0.83	0.81	1	8263	7957
cor(Intercept,Multiple)	0	0.38	-0.71	0.72	1	4569	5739
cor(Temp24,Multiple)	0.07	0.42	-0.76	0.80	1	4087	5397
cor(Temp28,Multiple)	-0.17	0.41	-0.86	0.67	1	4565	5899

~Host Phylogenetic (Number of levels: 7)

	Estimate	SE	lower CI	upper CI	Rhat	Bulk_ESS	Tail_ESS
sd(Intercept)	1.44	0.96	0.09	3.64	1.00	2977	4932
sd(Temp24)	1.03	0.61	0.08	2.45	1	3388	3718
sd(Temp28)	2.64	1.67	0.28	6.61	1	4841	3671
cor(Intercept,Temp24)	0.03	0.47	-0.83	0.86	1	5740	6879
cor(Intercept,Temp28)	-0.18	0.46	-0.89	0.77	1	6743	6462
cor(Temp24,Temp28)	-0.1	0.47	-0.9	0.77	1	8307	7863

Table S16. Summary of the Bayesian regression model used for the analysis of parasitism success of *Leptopilina sp*. alone or in multiple infection of different hosts. Results from a multilevel model including temperature, infection type and their interaction as categorical fixed parameters and two grouping variables: host as a categorical variable with temperature and infection type as group-level variables, and host accounting for phylogenetic correlations with temperature included as a group-level variable. Models were built using the R package *brms*, with eight chains, each with 1250 iterations after the 1000 iteration warmup, totalling 10^4 posterior samples. Estimate: mean of the posterior distribution samples, SE: Standard error of the estimate, calculated as the standard deviation of the posterior samples. lower and upper CI: lower and upper 95% credible intervals, also obtained from the posterior distribution. Rhat: the potential scale reduction factor on split chains (at convergence, Rhat = 1). Bulk and tail-ESS: diagnostic of autocorrelation in the posterior samples, the effective number of posterior samples at the bulk and tails of the posterior distribution. Rhat and ESS calculated following Vehtari et al. *(2021)*.

Fixed effect Coefficients:

	Estimate	SE	lower CI	upper CI	Rhat	Bulk_ESS	Tail_ESS
Intercept	-1.15	2.45	-5.72	3.92	1	4910	6201
Temp24	-0.11	1.39	-2.87	2.98	1	4701	4069
Temp28	-4.5	-<br>2.79	10.02	1.16	1	5306	4509
Multiple	-1.33	0.83	-3.08	0.27	1	5693	5577
Temp24:Multiple	0.4	0.15	0.11	0.69	1	16852	7682
Temp28:Multiple	-0.86	1.02	-2.97	1.03	1	13978	6674

Multilevel Hyperparameters

~Host (Number of levels: 7)

	Estimate	SE	lower CI	upper CI	Rhat	Bulk_ESS	Tail_ESS
sd(Intercept)	3.53	1.88	0.67	7.98	1	2949	3004
sd(Temp24)	1.13	0.82	0.07	3.17	1	3498	3290
sd(Temp28)	3.48	1.9	0.96	8.16	1	6024	4151
sd(Multiple)	1.75	0.75	0.82	3.72	1	5673	6428
cor(Intercept,Temp24)	-0.06	-<br>0.43	0.83	0.74	1	7544	7293
cor(Intercept,Temp28)	-0.29	-<br>0.38	0.89	0.53	1	6420	6532
cor(Temp24,Temp28)	0.2	-<br>0.39	0.61	0.85	1	6226	6983
cor(Intercept,Multiple)	-0.17	-0.4	0.86	0.63	1	5066	5097
cor(Temp24,Multiple)	0	-0.4	0.72	0.76	1	5295	6346
cor(Temp28,Multiple)	0.15	-<br>0.35	0.54	0.77	1	7583	7693

~Host Phylogenetic (Number of levels: 7)

	Estimate	SE	lower CI	upper CI	Rhat	Bulk_ESS	Tail_ESS
sd(Intercept)	3.41	2.49	0.19	9.53	1	2023	3939
sd(Temp24)	1.6	1.13	0.09	4.32	1	3119	4270
sd(Temp28)	2.26	2.24	0.08	7.9	1	4559	4355
cor(Intercept,Temp24)	0.13	0.48	-0.8	0.91	1	6609	5812
cor(Intercept,Temp28)	-0.09	0.5	-0.91	0.84	1	9249	7045
cor(Temp24,Temp28)	0.06	0.5	-0.86	0.89	1	8741	7751

Table S17. Summary of the Bayesian regression model used for the analysis of melanisation in *D. pseudotakahashii* by larval parasitoids. Results from a multilevel model including temperature as a categorical fixed parameter and parasitoid as a grouping variable. Temperature was also included as a group-level variable for parasitoids. Models were built using the R package brms, with eight chains, each with 1250 iterations after the 1000 iteration warmup, totalling 10^4 posterior samples. Estimate: mean of the posterior distribution samples, SE: Standard error of the estimate, calculated as the standard deviation of the posterior samples. lower and upper CI: lower and upper 95% credible intervals, also obtained from the posterior distribution. Rhat: the potential scale reduction factor on split chains (at convergence, Rhat = 1). Bulk and tail-ESS: diagnostic of autocorrelation in the posterior samples, the effective number of posterior samples at the bulk and tails of the posterior distribution. Rhat and ESS calculated following Vehtari et al. *(2021)*.

Fixed effect Coefficients:

	Estimate	SE	lower CI	upper CI	Rhat	Bulk_ESS	Tail_ESS
Intercept	4.26	2.13	0	8.72	1	4408	5355
Temp	-0.17	0.12	-0.4	0.09	1	3956	4202

Multilevel Hyperparameters

~Parasitoid

	Estimate	SE	lower CI	upper CI	Rhat	Bulk_ESS	Tail_ESS
sd(Intercept)	3.74	1.78	1.39	8.21	1.00	4597	5860
sd(Temp)	0.28	0.18	0.08	0.77	1.00	2539	4208
cor(Intercept,Temp)	-0.41	0.4	-0.95	0.52	1.00	3519	4988

	odds.ratio	lower.HPD	upper.HPD
A / C	5.450	3.49	8.000
G / C	2669.190	24.4	75194075
L / C	4.000	2.630	6.000

	odds.ratio	lower.HPD	upper.HPD
(A / C) / (G / C)	0.002	0.00	0.037
(A / C) / (L / C)	1.364	0.972	1.834
(G / C) / (L / C)	656.700	7.743	19580000

	odds.ratio	lower.HPD	upper.HPD
Temp20/Temp24: A/C	0.975	0.39	1.753
Temp20/Temp24: G/C	0.259	0.000	1.362
Temp20/Temp24: L/C	0.340	0.146	0.653

Table S18. Summary of the Bayesian regression model used for the analysis of melanisation in *D. pseudotakahashii* by pupal parasitoid. Results from a model including temperature as a categorical fixed parameter and parasitoid treatment as a grouping variable. Temperature was also included as a group-level variable for parasitoid treatment. Models were built using the R package brms, with eight chains, each with 1250 iterations after the 1000 iteration warmup, totalling 10^4 posterior samples. Estimate: mean of the posterior distribution samples, SE: Standard error of the estimate, calculated as the standard deviation of the posterior samples. lower and upper CI: lower and upper 95% credible intervals, also obtained from the posterior distribution. Rhat: the potential scale reduction factor on split chains (at convergence, Rhat = 1). Bulk and tail-ESS: diagnostic of autocorrelation in the posterior samples, the effective number of posterior samples at the bulk and tails of the posterior distribution. Rhat and ESS calculated following Vehtari et al. *(2021)*.

Fixed effect Coefficients:

	Estimate	SE	lower CI	upper CI	Rhat	Bulk_ESS	Tail_ESS
Intercept	5.64	2.31	1.23	10.43	1.00	3785	2934
Temp	-0.29	0.13	-0.57	-0.03	1.00	2102	977

Multilevel Hyperparameters

~Parasitoid

	Estimate	SE	lower CI	upper CI	Rhat	Bulk_ESS	Tail_ESS
sd(Intercept)	1.66	1.66	0.04	5.97	1	2788	3214
sd(Temp)	0.2	0.29	0	0.99	1	1669	1740
cor(Intercept,Temp)	-0.16	0.59	-0.98	0.93	1	3607	4073

	odds.ratio	lower.HPD	upper.HPD
T / C	1.180	0.40	2.410

	odds.ratio	lower.HPD	upper.HPD
Temp20/Temp24: T/C	0.993	0.362	1.810

Supplementary References

Vehtari, A., Gelman, A., Simpson, D., Carpenter, B. & Bürkner, P.-C. (2021). Rank-Normalization, Folding, and Localization: An Improved Rˆ for Assessing Convergence of MCMC (with Discussion). *Bayesian Anal.*, 16.
